# Auxin controls circadian flower opening and closure in the waterlily

**DOI:** 10.1186/s12870-018-1357-7

**Published:** 2018-07-11

**Authors:** Meiyu Ke, Zhen Gao, Jianqing Chen, Yuting Qiu, Liangsheng Zhang, Xu Chen

**Affiliations:** 1 College of Horticulture and Fujian Provincial Key Laboratory of Haixia Applied Plant Systems Biology, Fujian Agriculture and Forestry University, Fuzhou, Fujian China; 2 FAFU-UCR Joint Center for Horticultural Biology and Metabolomics, Haixia Institute of Science and Technology, Fujian Agriculture and Forestry University, Fuzhou, 350002 China

**Keywords:** Waterlily, Flower opening and closure, Auxin, Transcriptome

## Abstract

**Background:**

Flowers open at sunrise and close at sunset, establishing a circadian floral movement rhythm to facilitate pollination as part of reproduction. By the coordination of endogenous factors and environmental stimuli, such as circadian clock, photoperiod, light and temperature, an appropriate floral movement rhythm has been established; however, the underlying mechanisms remain unclear.

**Results:**

In our study, we use waterlily as a model which represents an early-diverging grade of flowering plants, and we aim to reveal the general mechanism of flower actions. We found that the intermediate segment of petal cells of waterlily are highly flexible, followed by a circadian cell expansion upon photoperiod stimuli. Auxin causes constitutively flower opening while auxin inhibitor suppresses opening event. Subsequent transcriptome profiles generated from waterlily’s intermediate segment of petals at different day-time points showed that auxin is a crucial phytohormone required for floral movement rhythm via the coordination of YUCCA-controlled auxin synthesis, GH3-mediated auxin homeostasis, PIN and ABCB-dependent auxin efflux as well as TIR/AFB-AUX/IAA- and SAUR-triggered auxin signaling. Genes involved in cell wall organization were downstream of auxin events, resulting in the output phenotypes of rapid cell expansion during flower opening and cell shrinkage at flower closure stage.

**Conclusions:**

Collectively, our data demonstrate a central regulatory role of auxin in floral movement rhythm and provide a global understanding of flower action in waterlily, which could be a conserved feature of angiosperms.

**Electronic supplementary material:**

The online version of this article (10.1186/s12870-018-1357-7) contains supplementary material, which is available to authorized users.

## Background

In flowering plants, many flowers open during the day and close at night, exhibiting a 24-h circadian rhythm, which is known as Linné’s floral clock [1]. This circadian movement pattern serves as an environmental adaptation characteristic for creating an opportunity for pollination [1]; in *Ipomoea nil*, the flower-opening time is determined by the length of dark period [2]. However, without a light-dark switch period, flowers continue to display a rhythm in opening and closing when in a complete light or dark environment using their “internal clock”, i.e. circadian clock [3]. Yon et al. found that silencing the core circadian clock genes Late Elongated Hypocotyl (LHY) and ZEITLUPE (ZTL) in *Nicotiana attenuate* strongly altered the floral rhythms [4]. Therefore, the internal circadian clock can recognize a 24-h rhythmic oscillation, establishing an endogenously sensing system to adapt to seasonal environmental cycles. Due to the daily changes in solar direction and irradiation, light becomes the key connection between the circadian clock and other environmental signaling pathways. So far, sophisticated light responsive networks have been intensively established in model plant developmental processes (summarized in [5]), and the output models are conceptually useful for explaining the connections between light and circadian rhythm in flowers. For example, sunflowers track the sun from east to west, reorienting during the night so that their leaves and apices face east before sunrise [6]. We term this phenomenon as phototropism, which is a typical plant response to light identified during floral rhythms [7]. Phototropism has been well characterized as being controlled by an asymmetric auxin distribution [8]. Correspondingly, application of the auxins indole acetic acid (IAA) and naphthaleneacetic acid (NAA) strongly promotes the opening of iris flowers [9, 10]. Thus, auxin is also involved in the regulation of circadian floral movement.

Auxin, one of the major plant hormones, is essential to regulate diverse processes of plant development by coordination of cell expansion and cell division, such as embryogenesis, tropic response, shoot branching, vascular development, apical dominance, flowering and fruit ripening [11, 12]. The effects of auxin on plant development have been largely attributed to the transcriptional and translational regulations governed by the tryptophan aminotransferase (TAA) and flavin monooxygenase (YUC)-dependent synthesis pathway, Transport Inhibitor Response1/Auxin-Related F-BOX (TIR1/AFB)-Aux/IAA-regulated signaling pathway and PIN-FORMED (PIN)-mediated transport pathway [13–18]. Cell wall biogenesis has been implicated as a core event downstream of auxin regulations that determines anisotropic cell expansion during plant development including floral circadian movement. In *Ipomoea nil,* transcripts of xyloglucan endotransglucosylase/hydrolases (XTHs) encoding the genes InXTH1-InXTH4 in petals, which are involved in cell wall modification, were closely correlated with the rate of flower opening, further controlling plastic petal growth [2]. In the ‘Mitchell’ petunia, knockdown of cell-wall-associated β-galactosidases, which determine the galactose level among cell wall polysaccharides, severely reduces the flower-opening angle due to the disruption of petal integrity [19]. Hence, a combination of transcriptional, translational and post-translational regulations leads to coordination among various processes including the circadian clock, hormone biosynthesis, light signaling, and cell wall modification to establish a proper opening-closing period in flowers. Although the phenomenon of floral movements have been broadly described during the past 20 years’ research [9, 20], the underlying regulatory mechanisms are largely elusive.

Flower is a characteristic feature of the angiosperms, and floral rhythm has long been discussed regarding the evolutionary origin of flower and subsequent diversification. *Nymphaeales* (also called waterlily), one of the most ancient angiosperm lineages, represents an early-diverging grade of flowering plants [21]. *Nymphaeales* consists of five genera with approximately 60 species [22, 23], and most waterlily species have flowers that open during the day and close at night. Thus, owing to the special phylogenetic position of *Nymphaeales,* evaluating flower actions in *Nymphaeales* is highly useful to reconstruct the molecular characteristics of angiosperms.

In this study, *Nymphaeales* flowers were used as a model to study the rhythm of floral movement. We observed that the intermediate segment of petal cells of the *Nymphaeales* flower are severely flexible and respond to the photoperiod. Exogenous application of auxin pronouncedly blocks flower closure; whereas, treatment of auxin inhibitor suppresses flower opening. Transcriptome analysis generated from the intermediate segment of petals at different day-time points provide a global understanding that auxin metabolism, signaling and transport coordinate the movement rhythm formation of flowers. In addition, cell wall modification which acts as a downstream cascade of auxin is synchronously oscillating with auxin events, resulting in circadian cell expansion to accomplish floral opening and closing. This study offers an integrated view for understanding floral circadian rhythms and provides useful information for studying the traits of flowering plants across angiosperms.

## Results

### The petal is responsible for sensing the circadian signal for movement

**Fig. 1:**
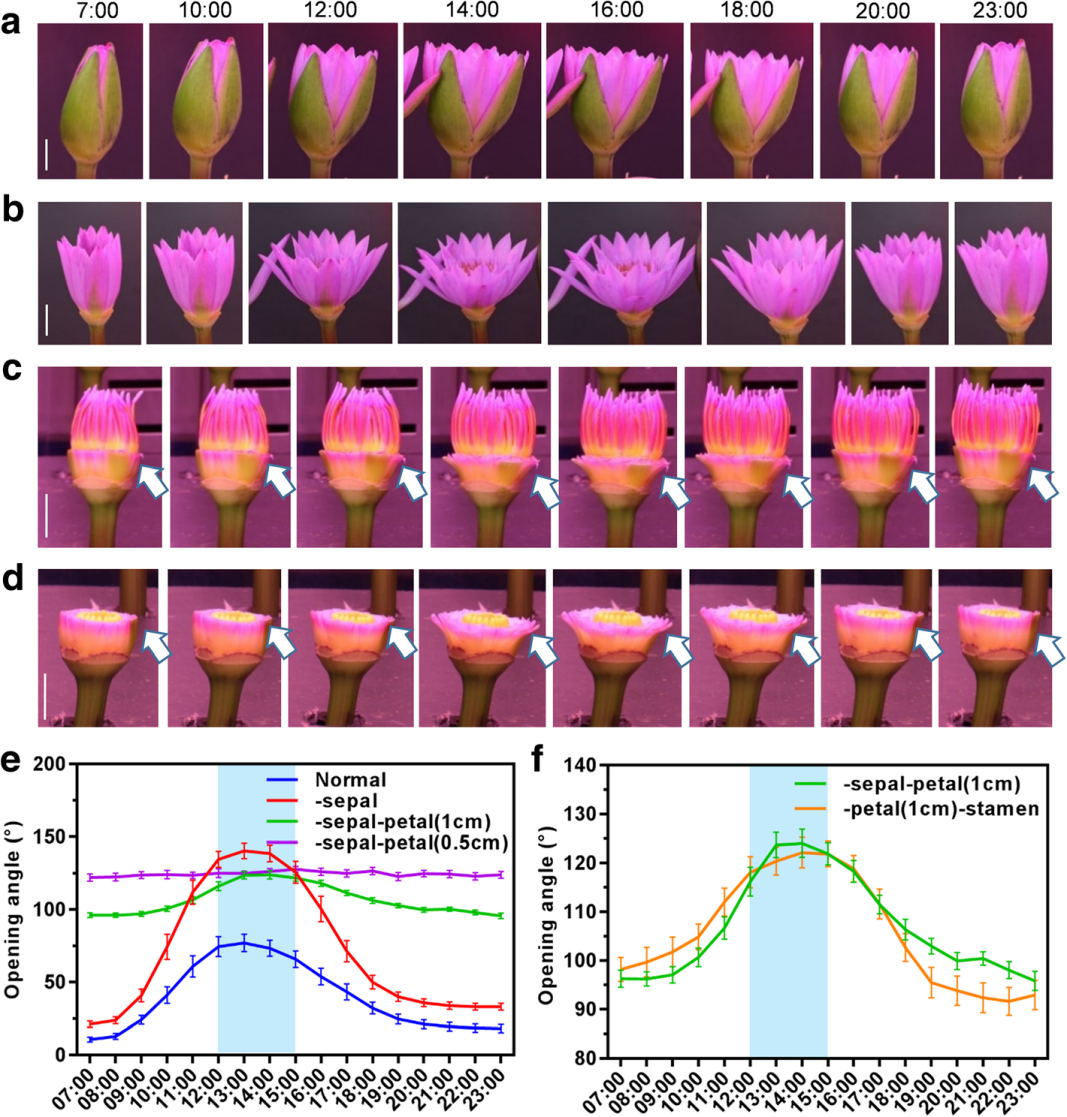
Intermediate petal determines flower opening and closure movement rhythm. **a** Floral opening and closure rhythm in *Nymphaea nouchali* cut flowers was tracked from 7:00 to 23:00. **b** Floral opening-closure rhythm without sepal. **c** Without sepal, the floral opening and closure rhythm was tracked when only 1 cm of the petal was left. White arrows highlighted the movement rhythm of the petals. **d** Without the sepal and stamen, the floral opening and closure rhythm was tracked when only 1 cm of the petal was left. White arrows highlighted the movement rhythm of the petals. **e**-**f** Floral opening-closure angles were measured according to the above pictures. Blue rectangular boxes below the curves highlight the maximum opening period. Scale bar, 25 mm (**a**-**d**). Error bar = SEM (**e**, **f**)

Plants keep track of the day-night photoperiod, ensuring reproductive success. To study this typical floral rhythm process and understand the mechanism by which the flower opening period is initiated, we adopted flowers from one of the most ancient angiosperm lineages, *Nymphaeales* (henceforth called waterlily), as the study model system. In the late summer of 2016 (photoperiod: 6:00–18:00 gradual light/18:00–6:00 gradual darkness; temperature: 25–32 °C), we tracked the flower movement of *Nymphaea colorata* via time-lapse photography for every 0.5 h. As shown in Additional file 1: Figure S1A, the flower remained closed at 7:30 and rapidly opened 0.5 h later (8:00); at 9:00, the opened angle of the flower reached the maximum value; and after 16:30, the flowers started closing continuously. Thus, the flowers of *Nymphaea colorata* showed a natural opening-closure pattern. Due to the shortage of *Nymphaea colorata* samples, we purchased the fresh-cut flowers of the waterlily cultivar *Nymphaea nouchali* as experimental materials. To test if the cut flowers exhibit a similar circadian movement pattern as natural flowers, the cut flowers were cultivated and tracked in a growth chamber at a controlled temperature at 25 °C and a 16-h light/8-h dark photoperiod (7:00–23:00 light/23:00–7:00 dark). Accompanied with the light exposure at 7:00, the flowers gradually opened. At 10:00, the opening angle rapidly increased until 14:00, when the opened angle reached the maximum of 75°. After 14:00, the flowers gradually closed, and at 23:00, the flowers were almost completely closed (Fig. 1a). Thus, with the manual manipulation of growth conditions, the cut flowers also displayed a stable opening-closure rhythm movement pattern, as shown by the key time points: 7:00 (initiation), 10:00 (rapid opening), 14:00 (maximal opening) and 18:00 (closure).

To explore the most sensitive part of flower that is responsive for period regulation, we first cut off the sepal of the flowers and tested the responsiveness between flowers with and without sepals. Without the sepals, the flowers continued to display an opening-closure pattern following period of (7:00–10:00–14:00–18:00) identical to that of the flowers with sepals. Moreover, without the sepals, the flowers exhibited a greater opening angle (maximum angle = 130°), suggesting that the sepals might act as a shield to restrict petal movement while protecting the internal flower organs (Fig. 1b, e). The sepals did not influence the floral rhythm; thus, we removed the sepals for the following experiments, owing to the convenience of study on internal flower organs.

To further evaluate the contribution of petals, the petals were divided into three sections: the top segment (> 1 cm distance to the bottom end), the intermediate segment (0.5–1 cm distance to the bottom end) and the bottom segment (< 0.5 cm to the bottom end). Petals without the top sections continued to response to the photoperiod and displayed a floral movement pattern similar to that of a complete flower (Fig. 1c, e), whereas the petals could not expand properly when only the bottom section remained (Fig. 1e, Additional file 1: Figure S1B, according to measurement standard of Additional file 1: Figure S1C). Meanwhile, the stamens exhibited an irreversible opening action (Fig. 1c, Additional file 1: Figure S1B). These observations suggest that the intermediate segment of petals is very sensitive to receive environmental signals and reacts. Furthermore, we even removed the stamens and left only 1 cm of the bottom segment of petals to verify if the stamens provide a thrust to push petal opening. However, without the stamens, the basal 1 cm segment of the petal was still able to respond to the photoperiod and display the 7:00–10:00–14:00–18:00 movement pattern (Fig. 1d, f). Thus, the physiological data indicated that the petal is the most important organ for receiving circadian signals and displays plastic curvature, and the 1 cm segment of the basal petal that connects to the sepal and the receptacle is primarily responsibility for floral movement.

### Cell morphogenesis of the intermediate petal is circadian-dependent

**Fig. 2:**
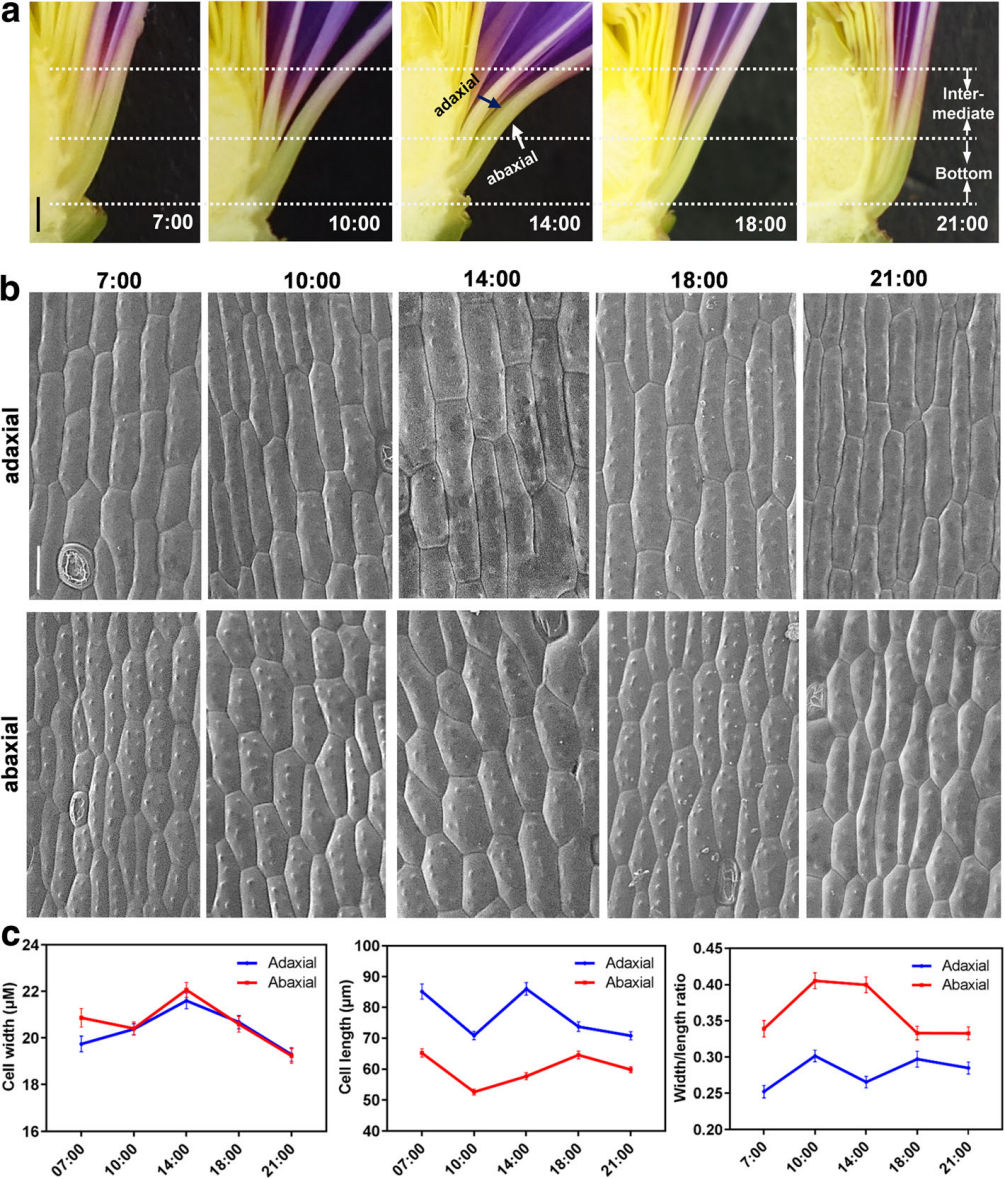
Petal intermediate cells are responsible for floral movement rhythm. **a** Longitudinal section of flowers showed that several petal layers were all responsive to the photoperiod. The adaxial and abaxial sides of the petals layers were marked, and white dot lines marked the intermediate and bottom segments of petals. **b**-**c** Cell morphology of the adaxial and abaxial epidermis at different time points was tracked by scanning electronic microscopy. Individual cell length and width, as well as the ratio of the cell width to the cell length, were quantified in the C chart. More than 85 cells were randomly selected and quantified in the individual samples. Scale bar, 2.5 mm (**a**), 25 μm (**b**). Error bar = SEM (**c**)

To determine whether the intermediate segment of petal is responsive for circadian rhythm, we tracked longitudinal sections of waterlily flowers at different time points (7:00, 10:00 and 14:00 were the flower-opening points, 18:00 and 21:00 were the flower-closure points). As shown in Fig. 2a, the intermediate segments of all four petal layers responded to the photoperiod, exhibiting gradually expanded curvatures. Particularly, the intermediate segment showed a strikingly plastic change accompanied with the circadian petal movement (Fig. 2a). To better visualize the cellular change, we used Scanning Electronic Microscopy (SEM) to track the adaxial and abaxial epidermis of the intermediate petal. In both the adaxial and abaxial epidermis, long and narrow cells quickly expanded or shrank along the horizontal direction at different time points. As we observed, from 7:00 to 14:00, the cells were continuously swelling, reaching a maximum cell width of ~ 22 μm; later, from 14:00 till 21:00, the expanded cells shrank back to 19 μm (Fig. 2b-c). Obviously, the amplitude of cell length alteration in adaxial side was higher than abaxial side. Moreover, flower opening (at 10:00) elevated adaxial cell width whereas did not influence abaxial cells, implying that adaxial cells were more flexible than abaxial ones. Hence, the cellular change of intermediate segment of petal was coordinated by the alteration of cell width and cell length (Fig. 2c). We thereby calculated a ratio to compare the cell width with its individual cell length; the abaxial cells apparently exhibited a circadian oscillation of width/length ratio, as shown by the highest at 10:00 and 14:00, when the flowers rapidly opened, and the lowest at 18:00 and 21:00, when the flowers were closing (Fig. 2c). This oscillation pattern of cellular morphogenesis is tightly correlated with petal curvature. Therefore, the rapid remodeling of cells confirmed the above speculation that the intermediate petal cells receive and respond to circadian movement signals, subsequently bending via anisotropic cell expansion.

### Flower opening-closure pattern is influenced by exogenous auxin

**Fig. 3:**
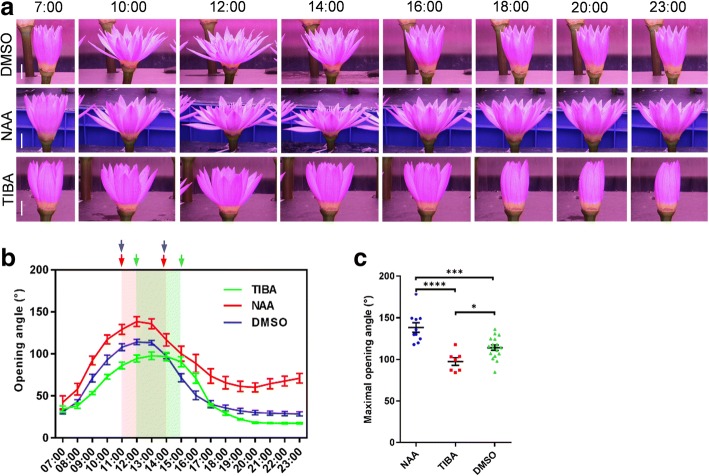
Flower opening-closure pattern is regulated by auxin. **a**-**c** Floral movement angles were measured after treatment with NAA and TIBA (10 μM NAA/TIBA was pretreated for 8 h before time-lapse photography, and then 10 μM NAA/TIBA was sprayed on the intermediate segment and bottom segments of petal for every 2 h treatment). Scale bar, 25 mm (**a**). Error bar = SEM (**b**, **c**). Significant analysis of the maximum opening angles among the DMSO-, NAA- or TIBA-treated samples was performed by Student’s t-test. ****, *p* < 0.0001

The plant hormone auxin is well-known to stimulate cell expansion and division via the promotion of cell wall flexibility and extensibility, which depends on the cell wall modification [24, 25]. Cell expansion at flower opening stage while shrinkage at flower closure stage are tightly correlated with the dynamic property regulated by auxin. It intrigues us to further understand if auxin is required for floral rhythm movement. To verify our speculation, we sprayed the synthetic auxin analog NAA and the auxin transport inhibitor 2,3,5-triiodobenzoic acid (TIBA) [26] on the intermediate petals before the phototracking. NAA significantly enhanced the rate and angle of flower opening (Fig. 3). Moreover, compared with the DMSO-control samples, whose closed angle was approximately 30°, the flowers could not be completely closed after NAA application, as shown by 70° closure angles at 20:00 and 80° at 23:00 (Fig. 3). It appears that NAA triggered the second rhythm of flower movement after 20:00. In contrast, TIBA suppressed flower opening. The maximum opening angle was significantly smaller than that in control, and the opening schedule was delayed for 1 h following TIBA treatment (Fig. 3). Thus, consistent with the role of auxin in the opening of the iris flower [10], our finding further unveils that auxin participates in the floral rhythm of opening and closure.

### Flower opening-closure pattern is coordinated by endogenous auxin metabolism

**Fig. 4:**
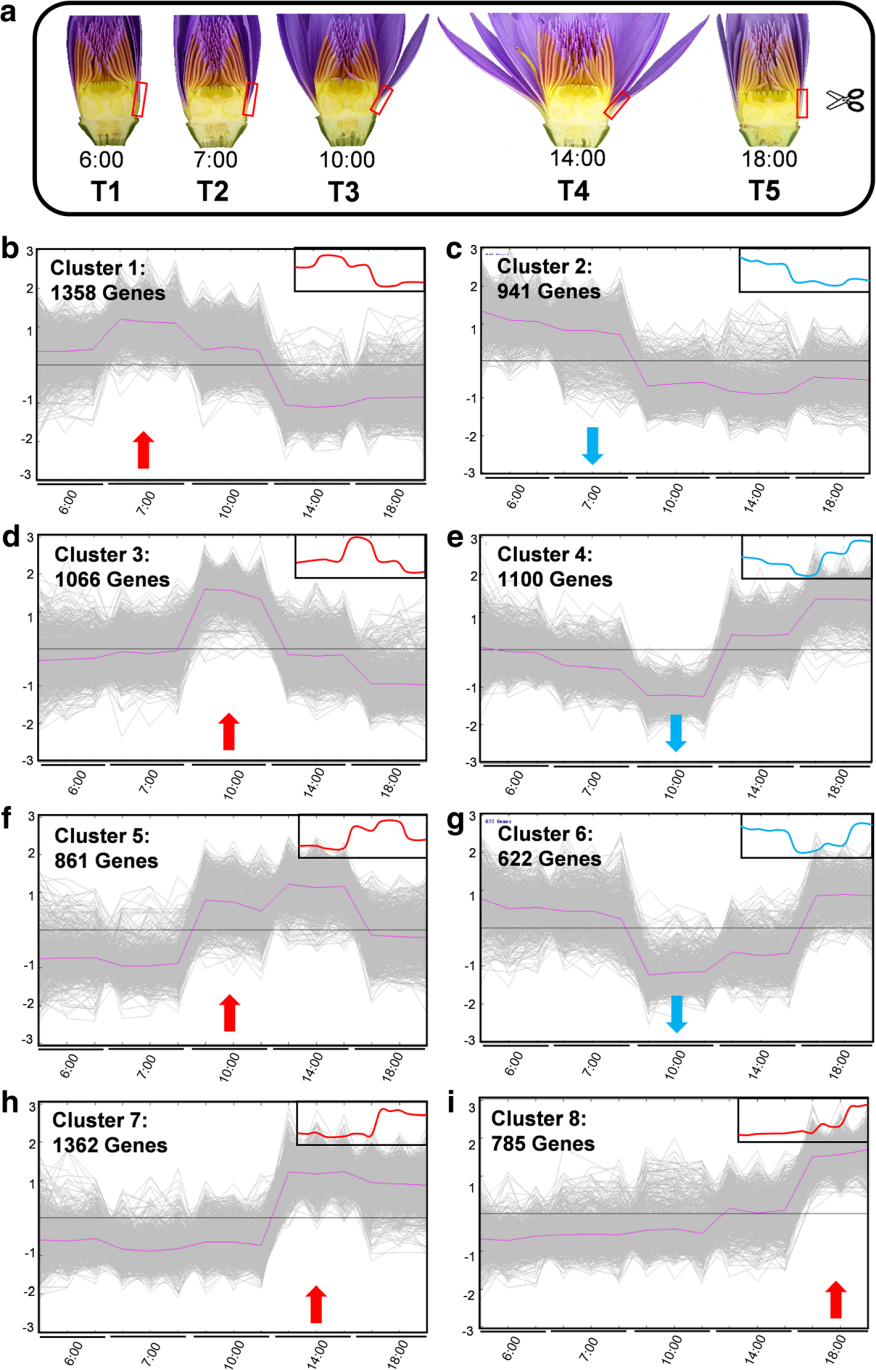
Global transcriptome description of flower opening-closure. **a** Description of RNA-seq transcriptome samples collected from the intermediate and bottom section of petals. **b**-**i** An overview of different clusters. The differentially expressed genes during the opening and closure of the flower at different time points (6:00, 7:00, 10:00, 14:00 and 18:00) were grouped into 8 clusters. The gene expression patterns of these 8 clusters is shown. Red arrows mark the clusters with upregulated genes, and blue arrows mark the clusters with downregulated genes. The global expression pattern curves were simulated in the upper right frames

In order to further understand the mechanism of auxin regulation and global transcription-based regulatory network occurred during the flower opening-closure processes, we performed a de-novo RNA-seq analysis based on the above described key time points of intermediate segment of petals. Samples at 6:00 (T1) were used as the control when the flowers were still under the dark period; and then, petal samples were individually collected at 7:00 (T2), 10:00 (T3), 14:00 (T4) and 18:00 (T5) (Fig. 4a). The differential transcriptome profiles were analyzed based on the standard of q-value< 0.05, fold change> 1.5, and FPKM (expected number of Fragments Per Kilobase of transcript sequence per Millions base pairs sequenced) ≥ 1 by comparison of different couples (such as T2 vs. T1). For annotation, in total 33,333 waterlily unigenes were searched and identified against the NCBI non-redundant (Nr) database using BlastX. According to GO enrichment and KEGG pathway analysis, in the T2 vs. T1 comparison group when the flowers started to receive the light signal, most of the downregulated genes (in total 422) were involved in oxidative stress and cell wall modification, and most of the upregulated genes participated in photosynthesis or lipid biosynthesis pathways (in total 166) (Additional file 2: Figure S2). At T3, the genes related to cell wall biogenesis started to be upregulated, corresponding to rapid flower opening action. At T4 when the flower opened maximally, the expression level of photosynthesis- and lipid-related genes decreased. When the flower was closing at T5, peroxidase-related genes were activated (Additional file 2: Figure S2). Thus, cells from the intermediate area of petals undergo a series of cellular changes in conjunction with the transcriptional regulation of photosynthesis, cell wall reorganization, oxidation, and lipid biosynthesis, among others.

To further understand the biological correlation during floral movement rhythm, we clustered all differential expressed genes into 8 clusters based on their expression pattern (Fig. 4b-i). At the onset of light stimulation at 7:00, the responsive genes of cluster 1 were immediately upregulated compared with their expression at 6:00 (Additional file 3: Figure S3A). According to a false discovery rate (FDR) < 0.05, 70 GO items of cluster 1 were found, and these items mainly belonged to photosynthesis and transmembrane transporters. Photosynthesis is a fundamental process used by plants to convert light energy to sugar and organic compounds [27]; therefore, photosynthesis I/II reaction center subunits and chlorophyll a-b binding proteins were greatly stimulated upon light stimulation (Additional file 3: Figure S3B, Additional file 4 Table S1).

**Fig. 5:**
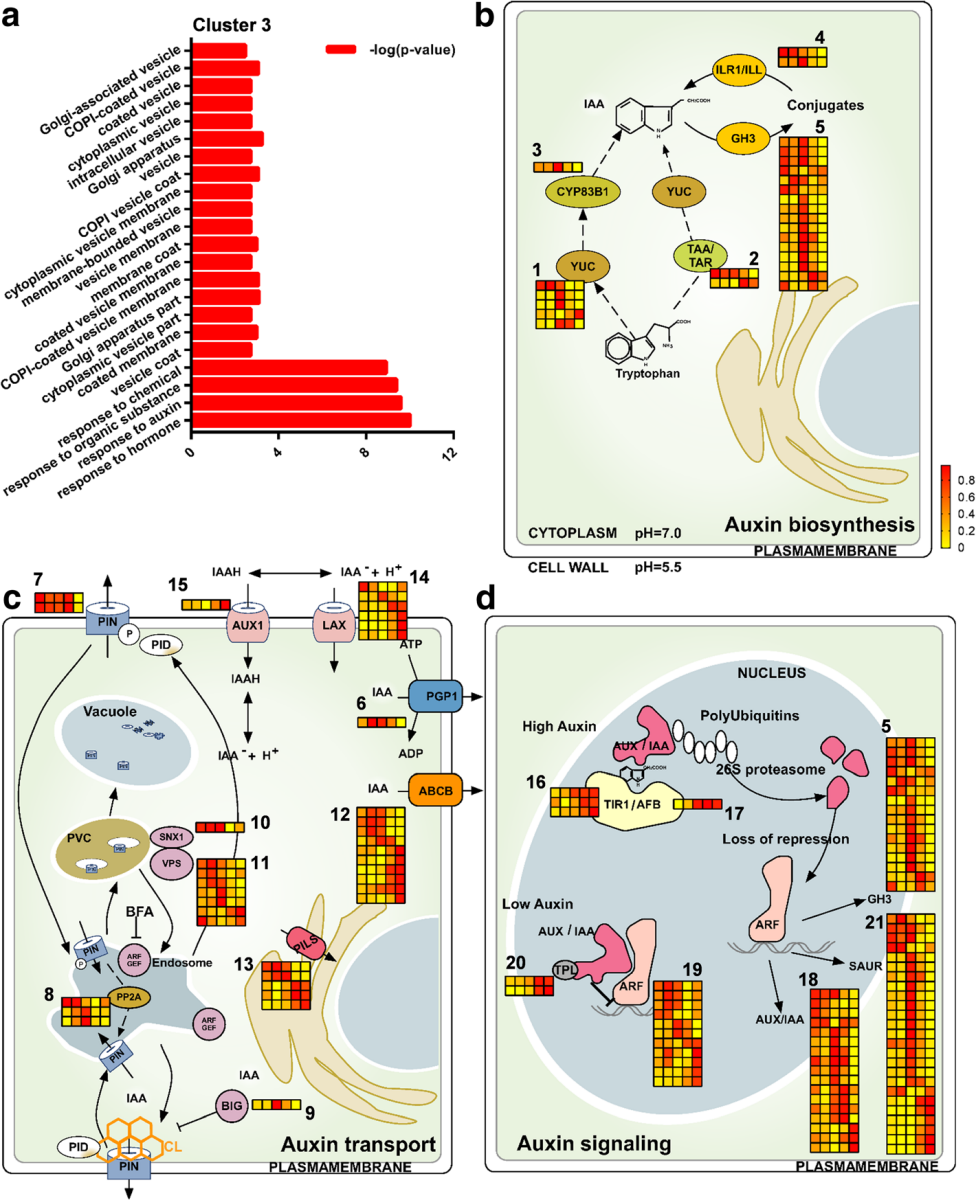
Flower opening-closure pattern is coordinated by auxin synthesis, signaling and transport. **a** GO items provided the transcriptome profiles of cluster 3, including vesicle trafficking, auxin signaling and cell wall modification-related genes. **b**-**d** Heatmap of individual regulators involved in auxin biosynthesis (**b**), auxin transport (**c**) and auxin signaling (**d**) provided the global transcriptome regulation of auxin during flower opening and closure processes (the corresponding genes were listed in Tables 1, 2 and 3 according to the labelled number “1” to “21” in each cluster) (modified from [89])

Based on the physiological data, opening of flower is regulated by exogenous auxin or auxin inhibitor treatment (Fig. 3), we try to understand if endogenous auxin level fine-tunes the regulation of floral movement rhythm. Indole-3-acetic acid (IAA) is the major natural auxin in plants and primarily synthesized from the substrate, Tryptophan (Trp). Trp is firstly converted to indole-3-pyruvate (IPA) by the Tryptophan aminotransferase (TAA) enzymes, subsequently IAA is produced from IPA by flavin monooxygenase (YUC) proteins [16, 28, 29]. Cytochrome P450 enzymes such as CYP72B2, CYP79B3 and CYP83B1 are also involved in auxin synthesis [15, 16]. Using annotated genes as query input, we tracked the DEGs of auxin metabolism events which were mainly present in cluster 3 (Fig. 5a-b). In the transcriptome profile, YUC homologues of *YUC8*, *YUC9* and *YUC10* were pronouncedly upregulated at the rapid opening stage of flowers and *YUC5* was activated at flower closure stage (Table 1, Fig. 5b). Similar as the oscillation of *YUCs*, *CYP83B1* was also induced at rapid opening stage. However, TAA family showed a different expression pattern, as shown by upregulation of *TAA3* but downregulation of *TAA2* during flower opening (Table 1, Fig. 5b). Nevertheless, most of auxin synthesis-related genes are activated at flower opening stage, indicating that floral movement rhythm requires the participation of new synthesized auxin (Fig. 5b).

**Table 1:** Auxin metabolism (corresponding to Fig. 5b)

Heat Map Number	Cluster Number	Gene ID	Best homolog	FPKM (6:00)	FPKM (7:00)	FPKM (10:00)	FPKM (14:00)	FPKM (18:00)
1	Cluster 1	DN47239_c2_g2	YUCCA8	53.1	59.0	42.6	7.5	12.2
	Cluster 3	DN35568_c0_g1	YUCCA9	9.6	8.3	44.6	13.5	5.2
	Cluster 3	DN35568_c0_g2	YUCCA8	29.1	29.4	167.1	51.1	20.1
	Cluster 4	DN37101_c0_g1	YUCCA5	18.6	17.3	5.6	26.4	53.8
	Cluster 5	DN30995_c2_g1	YUCCA10	5.0	4.1	12.7	11.6	4.0
2	Cluster 1	DN47222_c1_g2	TAA2	167.7	146.4	148.5	112.5	77.6
	Cluster 7	DN42557_c0_g1	TAA3	14.2	13.9	13.0	20.1	17.5
3	Cluster 3	DN28897_c2_g1	CYP83B1	191.6	191.8	268.3	170.5	137.8
4	Cluster 2	DN35005_c0_g1	ILR1-like 5	39.7	38.4	33.1	30.2	26.9
	Cluster 3	DN43770_c4_g2	ILR1-like 6	75.4	78.6	154.6	49.2	32.0
5	Cluster 1	DN46467_c1_g1	GH3.8	203.3	285.3	269.6	195.2	228.9
	Cluster 2	DN33284_c0_g4	GH3.8	20.8	17.0	8.4	8.4	9.0
	Cluster 3	DN39333_c1_g2	GH3.1	2.6	1.6	21.8	3.2	1.4
	Cluster 3	DN40159_c0_g3	GH3.6	3.2	3.6	7.2	1.8	1.1
	Cluster 3	DN37298_c3_g1	GH3.10	447.6	412.0	777.8	478.3	235.6
	Cluster 3	DN40159_c0_g1	GH3.5	4.3	6.5	18.5	6.6	2.3
	Cluster 3	DN39338_c0_g1	GH3.1	9.1	4.9	17.2	12.7	1.4
	Cluster 3	DN45718_c2_g1	GH3.10	290.4	356.5	747.9	431.1	222.9
	Cluster 3	DN35414_c0_g1	GH3.5	18.9	19.5	139.7	41.7	9.9
	Cluster 3	DN38188_c0_g1	GH3.17	16.3	10.2	111.1	51.7	1.1
	Cluster 3	DN35573_c3_g2	GH3.10	46.1	44.0	74.7	30.9	14.6
	Cluster 3	DN38138_c2_g2	GH3.1	86.8	55.3	94.9	39.1	10.8
	Cluster 3	DN39338_c0_g2	GH3.3	149.3	97.0	169.9	66.9	11.4
	Cluster 6	DN46059_c1_g1	GH3.8	131.2	102.6	87.3	106.5	113.8
	Cluster 7	DN39124_c2_g2	GH3.3	2.0	1.6	3.1	6.7	4.4
	Cluster 7	DN40159_c0_g2	GH3.6	22.1	14.0	14.8	21.7	16.0

Transcriptome profile of auxin metabolism-regulators, corresponding to Fig. 5b

Free IAA comprises no more than 25% of the total amount of IAA, depending on the plant tissue and species. Thus, in addition to free auxin, IAA is present in a variety of modified forms, including ester-linked indole-3-acetic acid (IAA)-sugar conjugates and amide-linked IAA-amino acid conjugates [30]. Free IAA is biologically active in diverse plant developmental processes; however, most IAA conjugates are inactive, and they serve as a reservoir to maintain appropriate balance of free IAA and conjugated IAA level [30]. The conversion of IAA to IAA-amino acids is determined by the Gretchen Hagen3 (GH3) family of auxin-inducible acyl amido synthetases [31]. In contract, IAA-Leu-resistant1/ILR1-like (ILR1/ILL) encodes an amidohydrolase that cleaves IAA-amino acid conjugates to release active IAA [32]. The transcriptome profile showed that *ILL5* and *ILL6* were downregulated along flower opening, whereas 11 out of 16 *GH3* homologues were particularly upregulated at flower opening stage (Table 1, Fig. 5b), suggesting that flower opening action triggers the frequent conversion of free IAA to conjugated IAA, which serves a storage of auxin for the further reuse. Hence, the influence of auxin synthesis and auxin homeostasis together demonstrated that flower opening triggers the transcriptional regulation of endogenous auxin metabolism, which coordinates the opening-closure rhythm formation of flower.

### Auxin flow toward the cell wall determines the plastic cell expansion

Auxin is synthesized in the tissues with active cell division and expansion, and is directionally transported via a cell-to-cell manner to the entire plant body [18]. The transportation of auxin is controlled by auxin influx and efflux carriers. PINFORMEDs (PINs) are the well-known auxin efflux transporters, which are constitutively cycling and recycling between the plasma membrane (PM) and endosomal compartments to control the polar auxin flow out of the cell [17]. Expression of *PIN1* homologues was relatively high at the flower opening stage while decreased when the flowers were closing (Fig. 5c, Table 2), suggesting that PIN-mediated auxin efflux is required for the rapid opening action. PP2A phosphatase is an important regulator for polar-targeting of PINs via dephosphorylation [33]. One *PP2A* homologue was downregulated while two *PP2A* homologues were upregulated during flower opening (Fig. 5c, Table 2), indicating that a fine-tune regulation of PIN phosphorylation occurs along the opening action. In addition, subcellular dynamics of PINs is coordinated by the endosomal regulators of guanine-nucleotide exchange factors for ADP-ribosylation factor GTPases (ARF-GEF) and Brefeldin A-inhibited guanine nucleotide-exchange protein (BIG) which mediate vesicle budding process [34–36], as well as Sorting Nexin1 (SNX1) and Vacuolar protein sorting proteins (VPS) which control vacuolar sorting process [37–39]. Expression of *BIGs* was stimulated associated with opening action, whereas *SNX1* and *VPSs* were downregulated at maximal opening stage (Fig. 5c, Table 2), implying that the opening of flower triggers frequent endocytosis events but suppresses degradation of PINs. Altogether, compared with flower closure stage, opening action induces PIN cycling between PM and endosomal compartments, thereby resulting in more auxin flow toward the cell wall.

**Table 2:** Auxin transport (corresponding to Fig. 5c)

Heat Map Number	Cluster Number	Gene ID	Best homolog	FPKM (6:00)	FPKM (7:00)	FPKM (10:00)	FPKM (14:00)	FPKM (18:00)
6	Cluster 1	DN34528_c0_g1	PGP1	43.7	72.7	66.8	49.9	28.4
7	Cluster 3	DN44477_c1_g1	PIN1	53.5	46.0	46.8	51.6	31.5
	Cluster 3	DN46442_c2_g2	PIN1	143.0	135.0	129.9	144.9	97.4
8	Cluster 2	DN43180_c1_g3	PP2A	64.2	63.2	49.0	41.8	52.9
	Cluster 3	DN37425_c3_g1	PP2A	86.9	108.1	126.6	83.9	88.2
	Cluster 3	DN40053_c1_g2	PP2A	60.4	71.8	85.0	63.4	60.5
9	Cluster 8	DN47566_c3_g2	BIG2	18.3	20.2	19.2	23.7	27.0
10	Cluster 1	DN34282_c0_g1	SNX1	58.8	59.8	60.5	49.0	52.8
11	Cluster 1	DN37945_c0_g1	VPS36	24.9	28.1	24.0	20.7	18.5
	Cluster 1	DN37945_c0_g2	VPS36	21.2	25.6	18.8	13.8	18.3
	Cluster 1	DN38531_c0_g1	VPS9A	14.0	16.5	15.9	12.9	11.8
	Cluster 3	DN30150_c0_g1	VPS25	25.9	22.9	48.8	20.6	14.1
	Cluster 3	DN33640_c2_g1	VPS37 homolog 2	107.1	115.5	142.2	98.1	105.0
	Cluster 4	DN35037_c3_g1	VPS2 homolog 2	3.0	2.3	1.9	4.1	5.6
	Cluster 6	DN38866_c1_g2	VPS28 homolog 2	94.3	68.3	55.7	63.5	81.2
12	Cluster 1	DN45340_c0_g1	ABCB28	28.2	37.2	36.1	24.7	20.2
	Cluster 1	DN41171_c0_g1	ABCB26	31.8	42.2	38.6	13.9	12.1
	Cluster 1	DN42867_c3_g1	ABCB26	28.3	43.5	36.4	14.4	14.4
	Cluster 3	DN42672_c0_g1	ABCB4	15.8	18.6	22.4	21.3	15.6
	Cluster 4	DN44388_c1_g1	ABCB1	55.5	58.2	34.8	67.5	99.2
	Cluster 4	DN44973_c0_g2	ABCB15	8.9	6.3	3.2	12.0	23.9
	Cluster 7	DN44285_c1_g2	ABCB2	15.4	16.2	21.1	43.2	47.2
	Cluster 7	DN32916_c0_g1	ABCB11	8.8	6.6	7.7	17.2	22.1
	Cluster 8	DN45326_c2_g6	ABCB20	17.8	21.1	26.3	29.2	32.9
	Cluster 8	DN41224_c0_g1	ABCB8	1.5	1.3	1.0	1.6	3.5
13	Cluster 1	DN41581_c1_g1	PILS6	15.4	17.2	14.3	8.9	8.6
	Cluster 1	DN40689_c0_g1	PILS2	47.2	56.8	60.6	34.2	32.2
	Cluster 7	DN42393_c0_g1	PILS7	43.8	28.5	49.1	81.8	68.6
	Cluster 7	DN42393_c0_g2	PILS7	70.5	39.4	78.0	122.2	105.4
	Cluster 8	DN33924_c0_g1	PILS3	2.1	2.3	2.9	4.4	5.6
14	Cluster 2	DN43979_c0_g6	LAX5	2.2	1.2	0.3	0.4	1.3
	Cluster 3	DN31179_c1_g1	LAX2	2.3	2.4	4.2	2.8	2.2
	Cluster 7	DN43979_c0_g2	LAX2	2.9	2.4	2.7	5.5	5.1
	Cluster 8	DN42551_c0_g1	LAX3	10.7	13.5	15.5	35.4	66.3
	Cluster 8	DN43979_c0_g1	LAX3	7.4	8.0	9.2	20.0	43.7
	Cluster 8	DN43979_c0_g3	LAX2	155.6	167.4	125.4	200.5	290.7
15	Cluster 4	DN36174_c1_g1	AUX1	104.0	97.2	70.3	128.5	186.9

Transcriptome profile of auxin transport-regulators, corresponding to Fig. 5c

P-glycoprotein (PGP/ABCB) proteins are defined as another type of auxin efflux transporters, which interact with PINs and maintain the stability of PINs at the PM [40]. Ten *ABCB* homologues were found in DEGs, and 6 of them were upregulated at rapid opening stage (Fig. 5c, Table 2), suggesting that ABCBs might assist PINs to maintain high auxin flowing out of the cell during rapid flower opening. Due to the sequence similarity with PIN proteins, PIN-LIKES (PILS) proteins are defined as the putative auxin carriers which regulate intracellular auxin accumulation and homeostasis at the endoplasmic reticulum (ER), subsequently affecting nuclear auxin signaling [41]. *PILS2* and *PILS6* were suppressed at maximal opening of flower, while *PILS3* and *PILS7* were promoted along flower opening (Fig. 5c, Table 2), indicating that floral movement stimulates a feedback auxin flow among the intercellular compartments.

Among all auxin transporters, the PIN and ABCB proteins are the major auxin efflux carriers, whereas AUXIN1/LIKE-AUX1 (AUX/LAX) proteins represent the major auxin influx carriers, which are responsive for auxin uptake within the cells [42]. Interestingly, 5 out of 7 *AUX1/LAX* homologue was upregulated at 14:00 and 18:00 of flower closing stage (Fig. 5c, Table 2), implying that auxin uptake is required for closure action. It is apparent that flower opening promotes auxin efflux while flower closing enhanced auxin influx. The above transcriptome data supported a scenario that flower opening action pumps more auxin to the cell wall, presumably leading to the further cell wall remodeling; while flower closing action keeps auxin stay within the cell, ready for the second round of floral movement.

### Auxin signaling is highly active throughout the entire floral movement rhythm

Auxin is extraordinarily multifunctional, with different cells responding very differently to changes in auxin levels, which is required for the coordination of auxin signaling at transcriptional level [43]. The core components of the nuclear auxin signaling machinery consist of three protein families: the F-box Transport Inhibitor Response 1/Auxin Signaling F-box Protein (TIR1/AFB) auxin co-receptors, the Auxin/Indole-3-Acetic Acid (Aux/IAA) transcriptional repressors, and the Auxin Response Factor (ARF) transcription factors [44]. Briefly, the small molecule auxin targets to its nuclear receptor Transport Inhibitor Response 1/Auxin Signaling F-BOX (TIR1/AFB) proteins, bringing TIR1/AFBs and transcriptional repressors Aux/IAAs together and subsequently leading to the ubiquitination and degradation of this complex. In the transcriptome profile, expression of *TIR1* and *AFBs* was gradually upregulated associated with floral movement rhythm, and reached to the maximal level at 18:00 (Fig. 5d, Table 3), suggesting that auxin signaling is required throughout the entire processes of flower opening and closure. Aux/IAAs act as transcriptional repressors to dimerize with Auxin Response Factor (ARF), allowing the rapid changes of transcription in response to auxin [28, 43]. In DEGs of waterlily Aux/IAA homologues, 13 out of 17 *IAAs* maintained high transcript during the opening process, but was suppressed at closing stage (Fig. 5d, Table 3). *TOPLESS (TPL)* which physically interacts with IAA12 and acts as a co-repressor to regulate auxin transcription [45], was consistently oscillating with *TIR1/AFBs* (Fig. 5d, Table 3). Moreover, the downstream components of auxin signaling, *ARFs* were also generally high expressed at flower opening stage (Fig. 5d, Table 3). Thus, expression of auxin signaling regulators was generally induced associated with flower opening, suggesting that opening action requires very active auxin perception network.

**Table 3:** Auxin Signaling (corresponding to Fig. 5d)

Heat Map Number	Cluster Number	Gene ID	Best homolog	FPKM (6:00)	FPKM (7:00)	FPKM (10:00)	FPKM (14:00)	FPKM (18:00)
16	Cluster 7	DN39076_c0_g1	TIR1	4.7	3.4	4.6	6.1	5.8
	Cluster 8	DN44053_c0_g1	TIR1	191.3	204.0	210.0	231.2	258.3
	Cluster 5	DN29985_c1_g2	TIR1	56.9	63.2	79.1	79.5	82.7
17	Cluster 5	DN44053_c0_g2	AFB2	39.5	43.3	52.4	53.7	51.8
18	Cluster 1	DN38861_c2_g1	IAA3	291.8	268.6	305.7	161.5	99.0
	Cluster 2	DN43322_c1_g2	IAA17	120.4	91.4	47.9	26.1	35.8
	Cluster 2	DN29291_c6_g2	IAA16	1378.1	1155.9	966.0	1013.8	880.1
	Cluster 3	DN36526_c1_g1	IAA14	97.8	64.2	142.4	98.5	21.4
	Cluster 3	DN41135_c1_g1	IAA31	16.7	11.7	49.2	12.8	2.1
	Cluster 3	DN39405_c0_g2	IAA3	426.1	332.4	654.6	537.9	263.5
	Cluster 3	DN36526_c2_g1	IAA30	750.4	511.0	817.6	784.3	293.3
	Cluster 3	DN42150_c0_g1	IAA26	13.5	9.4	18.7	11.7	4.0
	Cluster 3	DN36178_c2_g1	IAA13	7.9	7.9	19.2	8.6	3.6
	Cluster 4	DN40289_c0_g1	IAA26	4.6	3.8	2.6	5.4	6.9
	Cluster 4	DN33854_c1_g1	IAA25	42.3	30.2	11.2	33.5	97.7
	Cluster 5	DN41517_c0_g2	IAA8	507.9	417.5	523.9	579.1	482.1
	Cluster 5	DN35116_c0_g1	IAA11	12.9	8.3	31.5	41.5	10.6
	Cluster 5	DN41517_c0_g1	IAA9	711.7	611.9	763.7	808.9	683.0
	Cluster 5	DN43354_c0_g3	IAA8	300.1	293.1	350.9	386.0	265.8
	Cluster 6	DN39204_c2_g2	IAA33	27.3	28.7	13.3	26.8	23.5
	Cluster 8	DN40289_c0_g2	IAA7	43.0	47.3	10.9	44.2	186.9
19	Cluster 1	DN42573_c1_g3	ARF7	71.6	88.3	74.2	37.4	59.6
	Cluster 1	DN47912_c2_g2	ARF19	59.4	89.5	74.8	32.9	58.0
	Cluster 1	DN47912_c2_g1	ARF16	62.8	95.2	84.0	37.5	60.5
	Cluster 2	DN39252_c1_g1	ARF7	17.4	17.4	13.1	12.5	15.1
	Cluster 3	DN47227_c3_g1	ARF5	46.9	57.7	120.2	83.1	29.5
	Cluster 3	DN47227_c4_g1	ARF5	60.3	72.7	139.3	100.4	36.3
	Cluster 4	DN37116_c1_g1	ARF2	33.6	32.5	27.9	39.0	40.5
	Cluster 6	DN37212_c0_g1	ARF8	227.3	298.4	232.6	199.7	322.0
	Cluster 6	DN41045_c0_g1	ARF2	14.6	17.3	12.3	13.2	19.4
	Cluster 8	DN44980_c1_g1	ARF18	7.0	7.1	6.6	8.8	12.4
	Cluster 8	DN40890_c1_g1	ARF5	0.5	0.6	0.6	1.1	1.6
20	Cluster 7	DN46701_c1_g3	TOPLESS1	14.8	15.9	19.4	30.0	28.3
	Cluster 7	DN47270_c1_g1	TOPLESS1	9.1	9.5	11.1	16.7	18.4
21	Cluster 1	DN34356_c1_g2	SAUR72	2.2	3.9	1.9	0.3	0.7
	Cluster 1	DN26029_c0_g1	SAUR36	36.2	33.2	39.4	21.0	8.5
	Cluster 1	DN30854_c2_g2	SAUR32	47.9	39.1	51.6	21.9	18.5
	Cluster 2	DN30854_c1_g1	SAUR36	67.4	67.9	24.8	16.1	36.8
	Cluster 3	DN41900_c0_g1	SAUR50	102.4	115.7	350.6	153.2	47.3
	Cluster 3	DN34089_c0_g2	SAUR64	5.1	4.6	25.9	10.2	1.6
	Cluster 3	DN35232_c0_g1	SAUR50	7.5	4.5	15.4	12.4	2.9
	Cluster 3	DN39216_c0_g2	SAUR66	3.3	2.1	22.8	8.6	1.5
	Cluster 3	DN42996_c6_g1	SAUR50	19.3	19.9	65.7	14.6	5.7
	Cluster 3	DN35909_c3_g1	SAUR50	65.9	51.3	186.8	81.6	30.3
	Cluster 3	DN34089_c0_g1	SAUR61	122.2	79.7	544.6	183.8	22.4
	Cluster 3	DN43134_c0_g1	SAUR36	455.9	334.9	2128.9	771.6	85.5
	Cluster 3	DN39216_c0_g4	SAUR62	10.7	5.8	70.1	28.0	2.3
	Cluster 3	DN47793_c0_g1	SAUR32	12.7	10.8	15.2	9.8	4.8
	Cluster 3	DN43134_c0_g2	SAUR36	6.8	8.0	46.2	15.1	2.3
	Cluster 3	DN41900_c0_g3	SAUR50	32.7	49.0	113.0	50.7	17.5
	Cluster 3	DN41900_c0_g2	SAUR50	68.8	74.2	236.7	52.8	17.3
	Cluster 5	DN25157_c0_g1	SAUR36	4.6	2.4	12.4	11.0	4.2
	Cluster 6	DN42146_c2_g1	SAUR71	47.4	61.2	9.8	15.6	50.9
	Cluster 7	DN26029_c1_g1	SAUR36	8.1	5.8	4.7	28.6	31.9
	Cluster 8	DN26314_c0_g1	SAUR36	2.5	3.1	3.3	22.5	34.0
	Cluster 8	DN27201_c0_g2	SAUR36	2.4	1.8	0.6	5.2	15.1
	Cluster 8	DN37811_c0_g1	SAUR71	0.8	0.4	0.2	2.9	5.1
	Cluster 8	DN26420_c0_g1	SAUR32	4.6	2.8	2.0	62.0	103.7
	Cluster 8	DN29435_c1_g1	SAUR32	1.3	0.7	0.9	8.3	17.1

Transcriptome profile of auxin signaling-regulators, corresponding to Fig. 5d

Particularly, DEGs of small auxin up RNA (SAUR) family are greatly found in our transcriptome profile, which represent the largest family of early auxin response genes with elusive auxin-related functions. Recently studies have implicated the key roles of SAURs which integrate auxin and environmental signals in a wide range of plant development, including auxin-dependent tropic growth, apical hook development, leaf senescence, root growth, seed germination etc. [46]. There are 81 SAURs in *Arabidopsis*, 58 SAURs in rice, 18 SAURs in moss, 134 SAURs in potato and 79 SAURs in maize [46]. Due to the gene evolution and differentiation, tandem and segmental duplication events are usually happened which contributed to the expansion of the SAUR family genes. Interestingly, 25 *SAUR* homologues were differentially expressed during floral opening-closure process: *SAUR32*, *SAUR36* and *SAUR72* were highly expressed at the onset of flower opening (7:00), while being suppressed at the later flower movement processes; 13 out of 25 *SAURs* were particularly induced associated with rapid opening process (10:00); and the rest of *SAURs* were stimulated at the closing stage (18:00) (Fig. 5d, Table 3). Thus, different SAUR members displayed distinct responsiveness to flower opening or closing actions, supporting the underlying roles of SAURs involved in auxin-mediated anisotropic cell expansion during floral movement.

In summary, through the regulation of auxin metabolism, auxin transportation and auxin signaling, auxin serves as a key phytohormone to determine the circadian movement rhythm of flowers.

### Cell wall remodeling is downstream of auxin events and controlled by auxin oscillation during floral movement

In addition to auxin regulations, plenty of vesicle trafficking-related genes were found in cluster 3, which maintained high expression levels from 7:00 till 10:00 (Figs. 4d, 5a, Additional file 5: Figure S4A), including multiple COPI vesicle coat genes such as *Coatomer Subunits*, *Conserved oligomeric Golgi complex subunits (COG)* and *Protein transport Sec genes* (Additional file 4: Table S2), suggesting that vesicle trafficking is the earliest responding process during the rapid opening stage. Vesicle trafficking is a dynamics and rapid movement process, including endocytosis and exocytosis, packaging the signal molecules from the synthesis locations, such as endoplasmic reticulum (ER) to Golgi body; the molecules are finally released to their locations either on the plasma membrane, secreted to the extracellular space or targeted to the vacuoles for degradation [47]. Particularly, vesicle flow is crucial for cellular morphogenesis as it delivers membrane cargoes and materials for cell wall formation. Thus, promotion of vesicle trafficking might activate reorganization of the cell wall components [48].

**Fig. 6:**
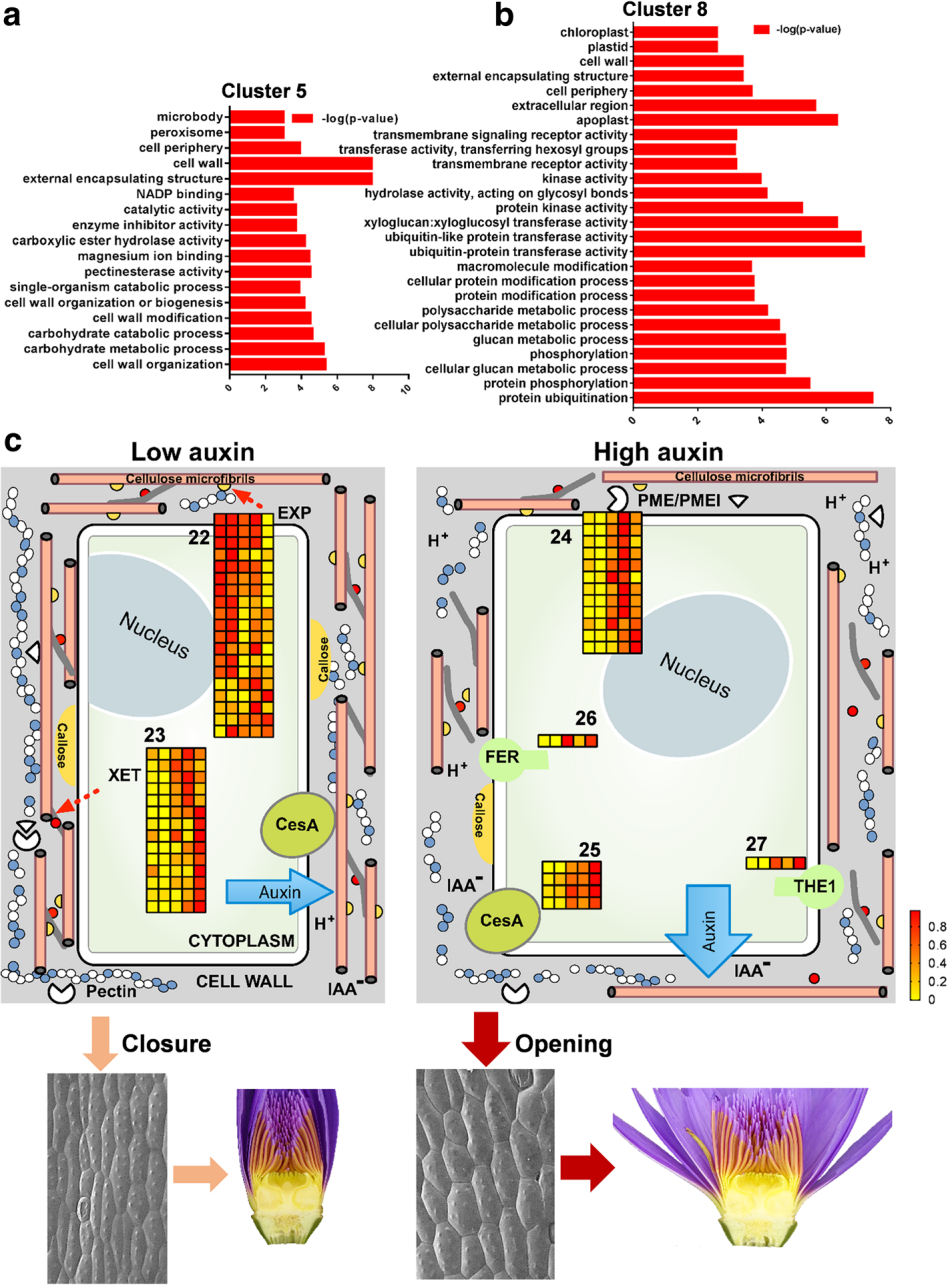
Cell wall remodeling is controlled by auxin during floral movement. **a**-**b** GO items provided the transcriptome profiles of cluster 5 and 8, including cell wall organization and modification, transmembrane receptor and kinase-mediated signaling cascades. **c** Heatmap of components involved in cell wall organization and modification provided the global transcriptome regulation of cell wall remodeling event, in conjunction of auxin regulation and floral movement rhythm (the corresponding genes were listed in Table 4 according to the labelled number “22” to “27” in each cluster)

Plant cell walls are rigid, flexible and extensible layers composed of polysaccharides, proteins, and lignin. Among the wall polysaccharides, cellulose, made of β-1,4-linked glucan microfibril, is the major wall component synthesized by cellulose synthase (CesA) complexes [49]. In the transcriptome profiles, multiple cell wall synthesis- or modification-related genes were differently responding to floral movement at distinct stages. For example, *CESAs* were gradually upregulated at 7:00 till 18:00, suggesting that cell wall is continuously synthesized along the entire floral movement process (Fig. 6c, Table 4). The synthesized cell wall components can be loosened either by cell wall-loosening proteins Expansin (EXP) or xyloglucan endotransglucosylase (XTH/XET). EXPs promote cell wall acidification to increase wall extensibility, and XETs break the xyloglucan cross-links between cellulose microfibrils to loosen cell wall [50, 51]. Several *EXPs* were highly expressed from 6:00 to 10:00 of the early stage of flower opening, whereas *XETs* showed upregulation from 10:00 till 18:00 (Fig. 6c, Table 4). Regarding the previous study showed that auxin triggers rapid cell wall acidification [52], the complementary responsiveness of *EXPs* and *XETs* to floral movement rhythm in our study also implies that EXPs-mediated wall loosening is important for early flower opening, probably in conjunction of auxin-induced rapid wall acidification; while the later stage of floral movement requires XETs-dependent cellulose microfibrils rearrangement.

**Table 4:** Cell wall synthesis (corresponding to Fig. 6C)

Heat Map Number	Cluster Number	Gene ID	Best homologs	FPKM (6:00)	FPKM (7:00)	FPKM (10:00)	FPKM (14:00)	FPKM (18:00)
22	Cluster 1	DN47643_c4_g1	Expansin-A4	6764.6	7055.4	5923.1	7291.2	2930.3
	Cluster 1	DN35711_c1_g1	Expansin-A8	571.1	548.8	447.6	516.6	193.8
	Cluster 1	DN39314_c1_g2	Expansin-A8	4679.1	4762.2	3940.4	4531.3	1636.7
	Cluster 1	DN28629_c0_g1	Expansin-B16	1.5	2.7	0.8	0.3	0.7
	Cluster 1	DN30671_c0_g2	Expansin-A2	185.6	178.1	165.4	159	89.9
	Cluster 2	DN35711_c1_g5	Expansin-A8	31.9	23.8	18.4	23.2	8.2
	Cluster 2	DN35711_c1_g5	Expansin-A8	31.9	23.8	18.4	23.2	8.2
	Cluster 2	DN30671_c0_g3	Expansin-A4	665.1	721	317.5	405.8	235
	Cluster 2	DN44906_c1_g1	Expansin-A13	5	6.3	1.7	3.3	4.1
	Cluster 2	DN33542_c0_g1	Expansin-A4	2532	2756.5	1432.3	2211.5	1727.9
	Cluster 2	DN33542_c0_g3	Expansin-A8	2025.5	2340.2	1052.4	1761	1436.1
	Cluster 2	DN30658_c1_g1	Expansin-A8	72.2	53.5	43.6	76	31.1
	Cluster 2	DN35711_c1_g2	Expansin-A8	993	1241	397.4	567	396
	Cluster 2	DN33542_c0_g3	Expansin-A8	2025.5	2340.2	1052.4	1761	1436.1
	Cluster 5	DN36402_c2_g1	PREDICTED:	574.8	646.8	1184.5	1763.4	873.6
			extensin-2-like					
	Cluster 4	DN29056_c1_g3	Expansin-A13	5.2	3.2	2.5	6.3	10.6
	Cluster 7	DN30658_c2_g4	Expansin-A6	814.8	876.9	738.4	1327.1	748.7
	Cluster 8	DN30658_c2_g9	Expansin-A11	0.9	1.1	1.9	2.5	3.3
	Cluster 8	DN30671_c1_g1	Expansin-A8	790.9	1119.3	960.3	939	1009.5
23	Cluster 8	DN29116_c0_g1	XET9	0.1	0	5.6	7.6	13.9
	Cluster 8	DN38195_c0_g3	XET15	1.1	0.1	2.6	1.3	11.1
	Cluster 8	DN38195_c0_g2	XET15	11.6	0.8	23	3.6	30.5
	Cluster 8	DN46820_c2_g2	XET24	1.3	1	3.1	20.6	36.3
	Cluster 8	DN31593_c1_g4	XET15	0.2	0.2	0.8	0.8	1.7
	Cluster 8	DN37190_c0_g1	XET23	16.2	3.2	6.6	5.6	32.5
	Cluster 8	DN39954_c0_g1	XET2	1.3	1	3.2	3	7.8
	Cluster 8	DN36023_c0_g1	XET8	65.3	66.8	57.3	81.7	126
	Cluster 8	DN35249_c0_g1	XET12	1.8	0.7	3.3	18.7	29.1
	Cluster 5	DN33376_c1_g1	XET33	63.8	50	70	94.8	74.4
	Cluster 5	DN43836_c1_g2	Probable XET	6711.1	8518.4	12501.5	10996.7	7990.8
	Cluster 5	DN43836_c1_g1	Probable XET-B	29.3	20.8	104.7	153.8	92.8
	Cluster 5	DN28612_c0_g1	XET7	36.1	41.4	108.5	199.8	76.1
	Cluster 5	DN35515_c1_g1	XET7	16.6	21	54	99.2	47.6
24	Cluster 8	DN37053_c0_g1	PME61	5.5	5.8	7.6	14.3	20
	Cluster 8	DN37066_c1_g2	PME34	5.8	6.2	7.9	14.4	21.4
	Cluster 5	DN38939_c0_g1	PPE8B	56.7	40.5	95.8	204.2	109.2
	Cluster 5	DN44786_c3_g2	PME40	25.1	21	71.1	128.2	62.6
	Cluster 5	DN42107_c2_g2	PME7	1.3	0.4	4.1	6.2	1.4
	Cluster 5	DN37053_c1_g2	PPE8B	54.4	36.3	89.3	185.7	99.4
	Cluster 5	DN40398_c2_g2	PME12	10.3	5	17.7	25.5	17.5
	Cluster 5	DN28149_c0_g1	PME7	6.8	8.4	28.9	23.3	6.4
	Cluster 5	DN38939_c0_g1	PPE8B	56.7	40.5	95.8	204.2	109.2
	Cluster 5	DN42067_c0_g2	Pectinesterase	20.5	15.2	51.2	90	46.4
	Cluster 5	DN46484_c1_g1	PME3	14.1	13.4	45.9	72.2	41.3
	Cluster 5	DN35306_c0_g1	PME68	38.9	34.4	51.3	41.5	46.1
25	Cluster 8	DN42344_c1_g1	CESA1	44.7	44.4	65.7	70.6	95
	Cluster 8	DN38997_c1_g2	CESA6	5.3	7.9	12.2	11.5	18
	Cluster 8	DN46153_c0_g1	CESA6	9.6	11.6	24	19.3	27.6
	Cluster 8	DN31684_c0_g1	CESA4	3.8	4.2	10.1	8	11.8
26	Cluster 8	DN40760_c0_g1	FERONIA	35.3	33.4	93.9	48	82.3
27	Cluster 8	DN43119_c1_g1	THESEUS 1	4	3.9	11.9	7.7	14.8

In addition, pectin is another major component of the plant cell wall, and it contains a highly heterogeneous group of polymers including homogalacturonans and rhamnogalacturonans I and II [53]. Pectinesterase (PME) catalyzes the de-esterification of pectin into pectate and methanol to alter the integrity of the cell wall [54]. Consistent with *XETs* expression, *PMEs* were additionally activated from 10:00 to 18:00 (Fig. 6c, Table 4), implying that a dramatic degradation and re-establishment of the cell wall occurred during the floral movement. Apparently, activation of these genes involved in cell wall remodeling was sustained throughout the entire process of floral movement, suggesting the importance of cell wall organization during flower opening and closure (Fig. 6c, Table 4).

Strikingly, a large number of plasma membrane-associated kinases, such as Serine/threonine-protein kinase, Brassinosteroid-Signaling Kinase 3 (BSK3) and NIMA (never in mitosis gene a)-related kinase 5 (NEK5) [55, 56]; Leucine-rich repeat receptor (LRR)-like serine/threonine-protein kinase, Receptor-like protein kinase 2 (RPK2) and Barely Any Meristem 1 (BAM1) [57, 58]; Receptor-like protein kinase FERONIA and transmembrane kinase (TMK) [59, 60], were found in cluster 8 (Additional file 5: Figure S4B, Additional file 4: Table S3). Most of them belong to plasma membrane-localized receptor-like kinases (RLKs). Ligands-activated RLKs are involved in a wide range of plant growth processes, such as plant immunity, male-female interaction and cell wall modification, which perceive and transmit extracellular signals into the cytosolic, leading to signaling transduction by phosphorylation [61–63]. For example, THESEUS1 (THE1) is a cell wall integrity sensor to mediate the response of plant cells to the perturbation of cellulose synthesis [64]. FERONIA (FER) elicits calcium transients to maintain cell wall integrity during salt stress [65]. In the transcriptome profile, *THE1* and *FER* were upregulated from 10:00 till 18:00 (Fig. 6c, Additional file 4: Table S3), which raised a possibility that RLKs might be the signal sensors of cell wall to coordinate the floral movement rhythm.

It has been well described that auxin induces cell elongation and expansion through its effect on modification of the cell wall [24]. The above transcriptome data further confirm that auxin signaling and cell wall remodeling were synchronously oscillating, leading to the output phenotype of cell expansion at flower opening stage and cell shrinkage at flower closure stage. Thus, we would speculate a scenario of floral movement rhythm: I. at flower opening stage when cells anisotropically expand, auxin is highly synthesized and transported to the cell wall, subsequently leading to the dramatic loosening and reorganization of cell wall; II. At flower closure stage when cell expansion are restricted, auxin synthesis is relatively delayed with low auxin flow to the cell wall (Fig. 6c). Thus, our finding further unveils that auxin-mediated cell wall remodeling is essential for the movement rhythm formation of flower opening-closure.

## Discussion

For the purpose of pollination, flowers open for a considerable period, and then abscise immediately after pollination [66]. Several factors are involved in this process, including transcriptional factors, hormonal regulation, cell wall modification and circadian rhythm control [9, 20]. In our study, one of the most ancient flowering plants, *Nymphaeales,* is used to explore the mechanism of floral rhythm. This work globally described auxin-dependent transcriptional signatures of flower opening-closure movement in *Nymphaeales*, which could be conserved in most of flowering plants.

Van Doorn et al. have summarized the major mechanisms during flower opening and closure [9, 20]. They concluded that turgor pressure and water transport are the major restrictive factors to limit fast movement. During the flower opening of the Asiatic Lily, the most influential factor is the change in angle between the midribs and the pedicel, where an increasing curvature of the midribs pulls the tepals apart [67]. Additionally, Liang and Mahadevana indicated that the edges of lily tepals are wrinkled as the flower opens [68]. Plant structures overcome these limits of turgor pressure, such as sepals of the waterlily flower, resulting in a very rapid cell expansion. Our research revealed that the intermediate petal is the major tissue responding to endogenous change and environmental stimuli. The realistic petal curvature determines the efficiency of insect-propagated pollination. Thus, plastic intermediate cells of petal offer a good platform to study the global signaling events during the circadian floral movement.

Consistent with the previous studies on light signaling which plays fundamental roles in diverse aspects of plant development, including floral movement rhythm, the crucial light signaling components were rapidly responding at the onset of flower opening (Additional file 3: Figure S3C). Using *Arabidopsis* homologues as input, the transcript oscillations of waterlily homologues of red/far-red light receptors Phytochromes (PHYs) [69], blue light receptor Cryptochromes (CRYs) [70] and Phototropins (PHOTs) [71], Ultraviolet-B receptor UV-B resistance 8 (UVR8) [72], and their downstream effectors including Phytochrome-Interacting Factor 3 (PIF3), Constitutive Photomorphogenic 1 (COP1), Suppressor Of Phytochrome A1 (SPA1), Elongated Hypocotyl 5 (HY5) [73] were depicted in a light signaling network (Additional file 3: Figure S3C, Additional file 4 Table S4). Moreover, the transcript level of the core circadian clock regulators, downstream of light signaling, such as Pseudo-Response Regulators (APRR) [74], Late Elongated Hypocotyl (LHY) [75] and Casein kinase (CKL) [76], were additionally triggered associated with flower opening (Additional file 3: Figure S3C, Additional file 4: Table S4). Therefore, the consistent upregulation of photosynthesis, light signaling and circadian clock events support the fundamental role of light at the beginning of flower opening.

Beside of the regulations of light signaling, auxin and cell wall, meanwhile other signaling pathways are significantly influenced associated with floral movement, as seen by downregulations of transcription factors and ribonucleoproteins in cluster 2, 4 and 6 (Additional file 6: Figure S5, Additional file 4: Tables S5-S7). In model plants, increasing numbers of TFs are shown to participate in flower development. For example, the expression of *ERF* TFs is highly correlated with flower senescence, because increased ethylene is produced during floral organ senescence [77, 78]. WRKY12 and WRKY13 oppositely regulate flowering under a short-day condition by controlling the plant hormone gibberellin [61]. Interestingly, RhNAC100 transcriptional factor was identified in rose petal, which is rapidly induced by ethylene in the petals. Silencing of RhNAC100 significantly increased the petal size and promoted cell expansion in the petal abaxial sub-epidermis [79]; this result is consistent with our finding that *NAC* TFs were downregulated during flower opening and petal cell expansion. Therefore, floral opening was associated with lower transcriptional cascades, suggesting that these transcriptional factors might act as negative regulators of cell expansion, which deserve further investigation.

## Conclusion

**Fig. 7:**
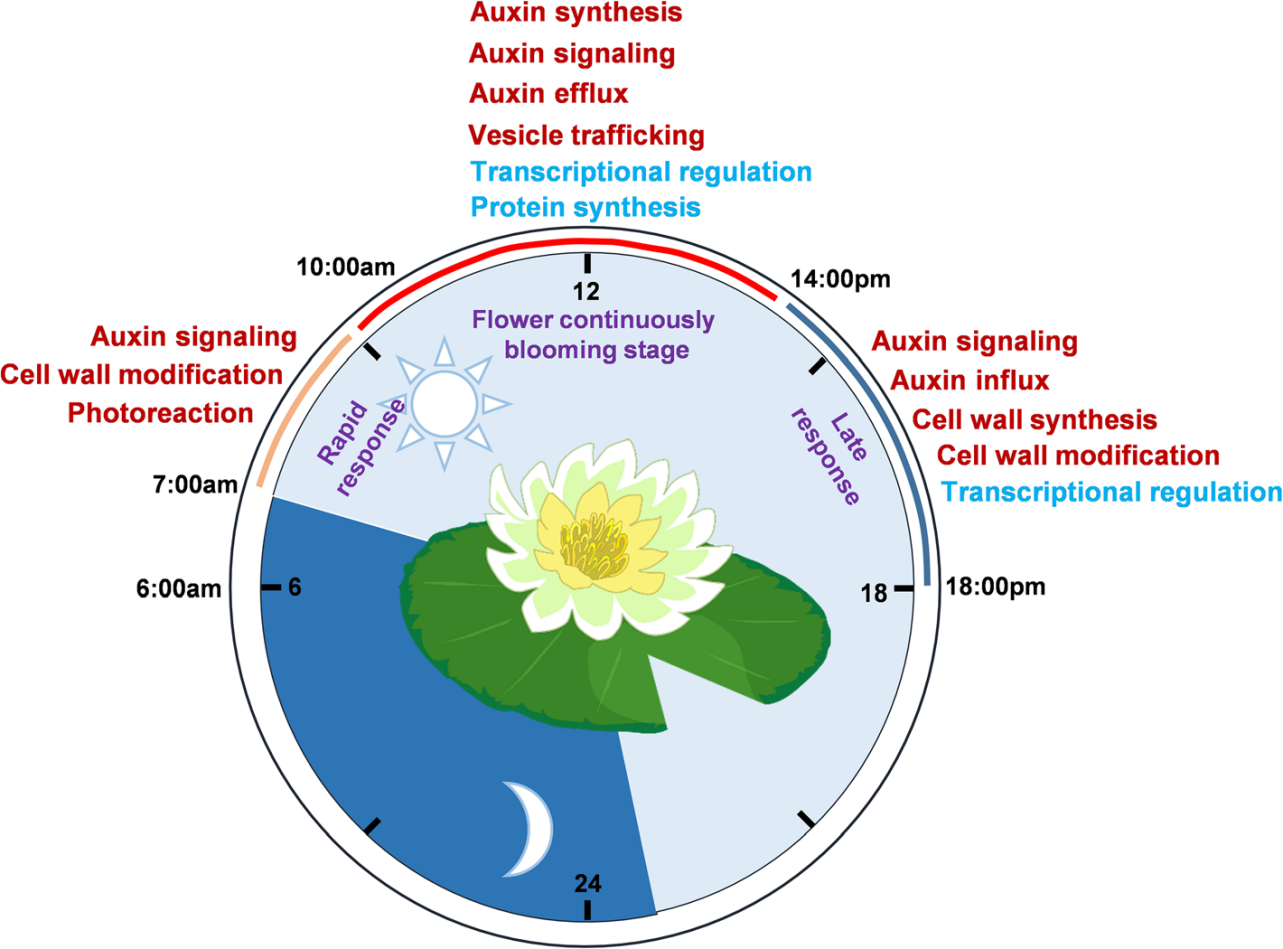
Preliminary model during flower opening and closure processes. During 7:00–23:00 light/23:00–6:00 dark photoperiod, the flowers gradually open from 7:00 to 14:00, and then followed by the closure process from 14:00 to 23:00. We divide the process into three stages: I. Rapid-response stage (7:00–10:00), involved in the upregulation of auxin signaling, cell wall modification and photoreaction events; II. Continuously blooming stage (10:00–14:00), involved in activation of auxin synthesis, auxin signaling, auxin efflux and vesicle trafficking; and suppression of transcriptional and translational events; III. Late-response stage (14:00–18:00), involved in the stimulation of processes including auxin signaling, auxin influx, cell wall synthesis and remodeling; and suppression of transcription. The upregulated pathways are marked red, and the downregulated pathways are marked blue

Similar to all touch responses, which can turn plants away from aggressors or enable flowers to move toward sunrise, morphogenesis during the flower opening-closure process is also influenced by a combination of endogenous and exogenous perturbations [80]. In our study, transcriptome profiles of the intermediate cells of petal were unearthed in the waterlily, which confirm that the signaling cascades of auxin metabolism, signaling and transport play center roles in flower opening and closure, and cell wall remodeling event regulated by auxin realizes circadian cell expansion and shrinkage associated with flower movement rhythm. Additionally, photosynthesis, light signaling and transcriptional regulations are individually incorporated into different stages of floral movement. Our study provided a preliminary understanding of the opening-closure processes of the waterlily flower by dividing them into three stages (Fig. 7).

Stage I (7:00–10:00): rapid-response stage, when the flowers are initially triggered for opening. At this stage, photosynthesis and light perception are activated, while gene transcription is blocked. Photosynthesis is the first reactive event once the flowers are exposed to light, which utilizes light energy to synthesize carbohydrate molecules (sucrose) for consumption or storage. Photoreception-mediated extension of the cell volume of the petal intermediate segment may be initiated by an unloading of sucrose from the phloem, possibly resulting in the lower apoplastic water potential [80], subsequently causing a change in cell turgor pressure. Active auxin perception system creates a complex but flexible environment, being able to re-arrange cell wall components (Fig. 7).

Stage II (10:00–14:00): flower-opening stage, when the flowers are quickly blooming until the maximal angle. At this stage, vesicle trafficking, auxin synthesis, auxin signaling and efflux, and cell wall remodeling processes occupy dominant roles during flowers blooming, accompanied with downregulation of transcription. Frequent vesicles trafficking events enhance the endocytosis and exocytosis of PM-located cargoes, such as auxin transporters PIN proteins, facilitating active auxin flow during organogenesis [81]. Apparently, auxin-related genes, such as GH3 family, AUX/IAAs, ARFs, SAURs are enriched in cluster 3 which particularly responded to rapid flower opening. YUC-dependent auxin synthesis, GH3-regulated auxin homeostasis, TIR1/AFBs and SAUR-mediated auxin signaling, as well as PIN- and PGP-regulated auxin efflux are primarily induced during stage II. A high level of auxin is synthesized and then being pumped to the cell wall; consequently, cell walls are acidified by high auxin and loosened by expansins [82].

Stage III (14:00–18:00): late response stage, when flowers start to be closed. The waterlily represents a typical flowering plant with a cross-pollination trait. The opened flower waits for insects that bring pollens from other flowers, crawling down towards its stigma for further pollination [80]. Apparently, the completely opened flowers with expanded stamens facilitate insects in touching the bottom of the stigma. Therefore, the fertilization event occurs usually after the maximum opening (after 14:00). In our transcriptome profiles, several RLKs are activated during stage III, possibly leading to fertilization. On the other hand, RLKs might function as cell-wall signaling receptors, in conjunction with cell-wall modification enzymes, to coordinate cell wall reorganization during flower closure. Auxin influx and cell wall modification events are still active in stage III, which confirms the crucial contributions of auxin and cell wall throughout the entire processes of flower opening and closure.

## Methods

### Plant materials and growth condition

*Nymphaea colorata* (Peter, Gustav Albert, 1928, Abhandlungen der Königlichen Gesellschaft der Wissenschaften, Göttingen. Mathematisch-Physikalische Klasse, n. s. 13(2): 58, 68, f. 10. 1928, website: http://www.tropicos.org/Name/22600193 or https://en.wikipedia.org/wiki/Nymphaea_colorata, preserved in Botanischen Garten, Berlin.) plants were cultivated under a natural environment, and the flower opening-closure pictures of *Nymphaea colorata* were captured in September of 2016 (Additional file 1: Figure S1A). For all the remaining experimental tests, cut flowers (with long stalks) of the waterlily cultivar *Nymphaea nouchali* (Burman, Nicolaas Laurens (Nicolaus Laurent), 1768, Flora Indica. .. nec non Prodromus Florae Capensis 120. 1768, website: http://www.ipni.org/ipni/idPlantNameSearch.do?id=135096-3&output_format=lsid-metadata&show_history=true, or https://en.wikipedia.org/wiki/Nymphaea_nouchali, preserved in Gandhi Park, East Fort, Thiruvananthapuram, India), which were commercially ordered from the market and hydroponically grown in a chamber controlled at 25 °C with a 16-h light/8-h dark photoperiod (7:00 am-23:00 pm light/23:00 pm-7:00 am dark), were used.

### Growth condition and chemical treatment

NAA (Biosharp) and TIBA (Macklin) were dissolved in dimethyl sulfoxide (DMSO). For the chemical treatment, 10 μM NAA and 10 μM TIBA were pre-sprayed on the intermediate and bottom section of the petals for 8 h before phototracking, and then the floral movement was tracked for the following 24 h by capturing images using an automatic camera.

### Time-lapse photography and measurement of flowering angles

The flower opening-closure patterns were tracked using time-lapse photography every 1 h for a total of 24 h. Deviated angles of petal opening and closure were measured using ImageJ (Additional file 1: Figure S1C). In each experiment, at least 10 flowers were analyzed, or at least 85 cells were measured. The experiments were repeated three times. Statistical analyses were performed using unpaired two-tailed t-test, where ns, *, **, *** and **** correspond to no significant difference, *p*-value < 0.05, < 0.01, < 0.001, < 0.0001, respectively.

### RNA-seq, expression annotation, GO and KEGG pathway enrichment analyses

The sepals of each flowers were removed, and the extracted samples were collected from the intermediate and bottom segments of petal (≤1 cm distance to the bottom end) at different times of 6:00, 7:00, 10:00, 14:00 and 18:00 (Fig. 3a). Each sample was collected from at least three flowers, and the samples at each time point were collected in triplicated and ground into fine powder with liquid nitrogen for RNA extraction. The total RNA of the waterlily flowers was extracted using the RNAprep Pure Plant Kit (Tiangen). RNA-Seq libraries were sequenced on an Illumina HiSeq X platform. After demultiplexing, the adapter sequences were trimmed, and poly-N and low-quality reads were removed to obtain the high-quality, clean reads. The Next Generation Sequencing (NGS) reads for the samples were listed in Additional file 4: Table S8. Trinity V2.06 was used to assemble the Paired-end clean reads and the assembled results indicated the contig Nx statistics (eg. the contig N50 value) (Additional file 4: Table S9). TransDecoder V2.0.1 was applied to predict the protein-coding regions of the transcripts. The NCBI/nr, SWISS Prot, and Phytozome V12 databases were searched to annotate the peptide functions [83]. To quantify the expression levels of genes, Trinity reference was annotated, and paired-end reads were mapped to the assembled transcripts and assigned to genes using RSEM V1.2.20. Differential expression analysis was performed using R package DEseq2 V1.18.1. The FPKM (expected number of Fragments Per Kilobase of transcript sequence per Millions base pairs sequenced) of each gene was calculated based on the length of the gene and the reads counts mapped to this gene. Genes with a fold change > 1.5 and a q value< 0.05 were assigned as differentially expressed genes (DEGs). The DEGs were mapped to GO terms in the GO database (http://www.geneontology.org/) in order to calculate the gene numbers for every term. KEGG (http://www.genome.jp/kegg/) is used to perform the pathway analysis of the DEGs. Statistical enrichment of the DEGs for the GO terms implemented by the agriGO v2.0. Significantly enriched GO terms and KEGG pathways (corrected *p* < 0.05) were identified based on a hypergeometric test [84, 85].

Cluster analysis was performed using the MeV4.8.1 software. Firstly, data were normalized by genes/rows. Secondly, significant DEGs were selected according to significance analysis for microarrays Delta = 0.26. The k-means method was used with the following settings: Pearson correlation, number of clusters = 8, and maximum iterations = 50 [86].

## Additional files

Additional file 1: The intermediate petal segment determines the flower opening and closure rhythm.Corresponding to Fig. 1. (A) The opening and closure rhythm of *Nymphaea colorata Peter*’s flower was tracked from 7:30 to 18:00. (B) Without the sepal, the floral opening-closure movement was tracked when only 0.5 cm of the petal was left. White arrows highlighted the petal movement. (C) Measurement methods of floral opening angles. Scale bar, 25 mm (A-C). (TIF 6190 kb)

Additional file 2: Global transcriptome description of the flower opening and closure. Corresponding to Fig. 4. Global analysis of GO items and KEGG pathways during the flower opening and closure processes are shown. The transcriptome at 6:00 (T1) was used as the control, and the transcriptomes at 7:00 (T2), 10:00(T3), 14:00 (T4) and 18:00 (T5) were individually compared with each other (T/T comparisons). The total changed numbers are listed below. Red represents the upregulated groups, and blue marks the downregulated groups (TIF 1165 kb)

Additional file 3: The involvement of light signaling during flower opening. (A-B) GO items and heatmaps provided the transcriptome profiles of cluster 1, including genes involved in photosynthesis (list in Additional file 4: Table S1). LHCB, Chlorophyll a-b binding protein; PSB, Oxygen-evolving enhancer protein; PSA, Photosystem I reaction center subunit V; CAB, Chlorophyll a-b binding protein; CAP, Chlorophyll a-b binding protein CP24; PPD, PsbP domain-containing protein; (C) Transcript profiling of light signaling network. The basic red/far-red light signaling cascade is based on the PHYs-PIF signaling module, as referred in [87]. Blue light signaling primarily consists of the CRY-COP1/SPA1-HY5 signaling module for photomorphogenesis, CRY-COP1/SPA1-GI-CO-FT module for photoperiodic flowering, PHOTs-mediated signaling for phototropism, as referred to [8, 70, 73]. UVB-triggered signaling is established by UVR8-COP1/SPA1-HY5 module, as referred in [72]. The input light signals additionally stimulate the endogenous circadian oscillator, which is feedback regulated by the transcriptional loop of the APRRs-TOCCCA1-LHY-ELF4-FKF1-ZTL clock signaling module, as referred to [88]. PHY, Phytochrome; CRY, Cryptochrome; PHOT, Phototropin; UVR8, UV-B resistance 8; PIF3, Phytochrome-interacting factor 3; ELF, Early flowering; COP1, Constitutive photomorphogenic 1; SPA1, Suppressor of phytochrome A1; HY5, Elongated hypocotyl 5; FKF1, Flavin Binding, Kelch Repeat, F-BOX1; ZTL, ZEITLUPE; APRR, Pseudo-Response Regulators; TOC1, Timing of CAB expression1; LHY, Late Elongated Hypocotyl; CCA1, Circadian Clock Associated 1; GI, GIGANTEA; CO, CONSTANS; FT, Protein Flowering Locus T; CKL, Casein kinase 1-like; EXP, Expansin. The expression patterns of these genes are marked as heatmaps (the corresponding genes were listed in Additional file 4: Table S4 according to the labelled number “28” to “42” in each cluster). (TIF 2690 kb)

Additional file 4: **Table S1.** Transcriptome profile of photosynthesis-regulators, corresponding to Additional file 3: Figure S3B. **Table S2.** Transcriptome profile of vesicle trafficking-regulators, corresponding to Additional file 5: Figure S4A. **Table S3**. Transcriptome profile of receptor kinases, corresponding to Additional file 5: Figure S4B. **Table S4.** Transcriptome profile of light signaling-regulators, corresponding to Additional file 3: Figure S3C. **Table S5.** Transcriptome profile of transcriptional factors, corresponding to Additional file 6: Figure S5B. **Table S6.** Transcriptome profile of protein synthesis-related genes, corresponding to Additional file 6: Figure S5D. **Table S7.** Transcriptome profile of transcriptional factors, corresponding to Additional file 6: Figure S5E. **Table S8.** NGS reads for waterlily RNA. **Table S9.** Statistical result of waterlily RNA transcriptome assembly. (DOCX 65 kb)

Additional file 5: Transcriptome description of vesicle trafficking and receptor kinase-mediated cascades. (A) Heatmap provided the transcriptome profiles of vesicle trafficking-related genes in cluster 3. The corresponding genes were listed in Additional file 4: Table S2. (B) Heatmap provided the transcriptome profiles of RLKs in cluster 8. The corresponding genes were listed in Additional file 4: Table S3. (TIF 640 kb)

Additional file 6: Description of flower opening-associated signaling events with downregulations. (A-F) GO items and heatmaps provided the transcriptome profiles of cluster 2, 4 and 6, mainly including transcriptional factors and protein synthesis-related genes (list in Additional file 4: Tables S5-S7). In B, ERF, Ethylene-responsive transcription factor; WRKY, WRKY transcription factor; NAC, NAC domain-containing protein; MADS, MADS-box transcription factor; HSFA, Heat stress transcription factor; BZIP, Basic leucine zipper; CDF, Cyclic dof factor. In D, RPL, ribosomal protein L; RPS, ribosomal protein S; TIF, Eukaryotic translation initiation factor. In E, Homeo-box, Homeobox-leucine zipper protein; Zinc finger, Dof zinc finger protein; ERF, Ethylene-responsive transcription factor; RAP, Ethylene-responsive transcription factor RAP; CRF, Ethylene-responsive transcription factor CRF; BBM, AP2-like ethylene-responsive transcription factor BBM; IDD, indeterminate-domain; NAC, NAC domain-containing protein; WRKY, WRKY transcription factor; GATA, GATA transcription factor; TGA, Transcription factor TGA. (TIF 3371 kb)
