# Supplementary figures and images for: Auxin controls circadian flower opening and closure in the waterlily
Source: BMC Plant Biol. 2018 Jul 11;18:143. doi: 10.1186/s12870-018-1357-7 (PMC6042438; doi:10.1186/s12870-018-1357-7)

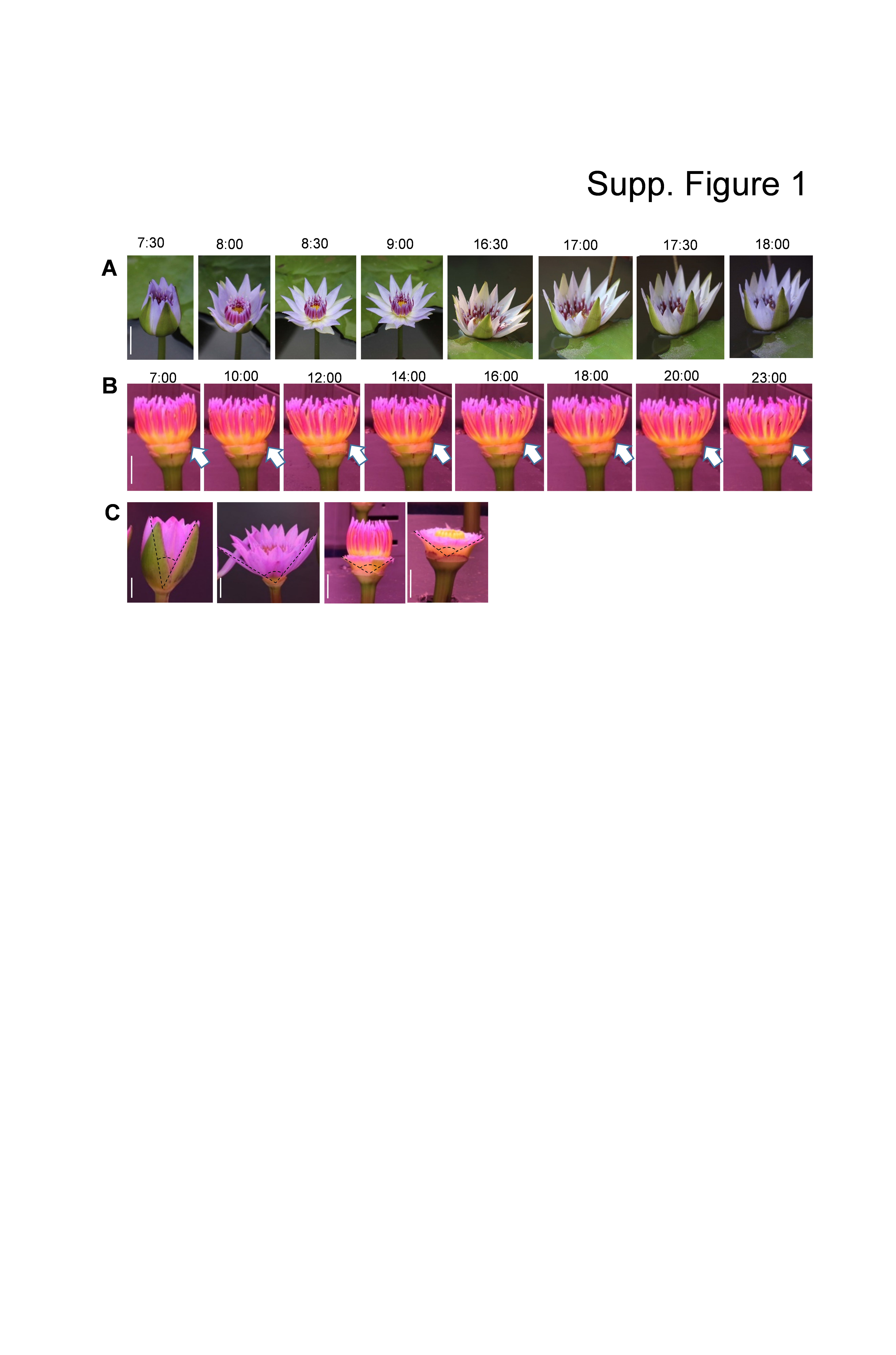

Supplement: Supplementary file 1 — The intermediate petal segment determines the flower opening and closure rhythm.Corresponding to Fig. 1. (A) The opening and closure rhythm of Nymphaea colorata Peter’s flower was tracked from 7:30 to 18:00. (B) Without the sepal, the floral opening-closure movement was tracked when only 0.5 cm of the petal was left. White arrows highlighted the petal movement. (C) Measurement methods of floral opening angles. Scale bar, 25 mm (A-C). (TIF 6190 kb) [file 12870_2018_1357_MOESM1_ESM.tif]

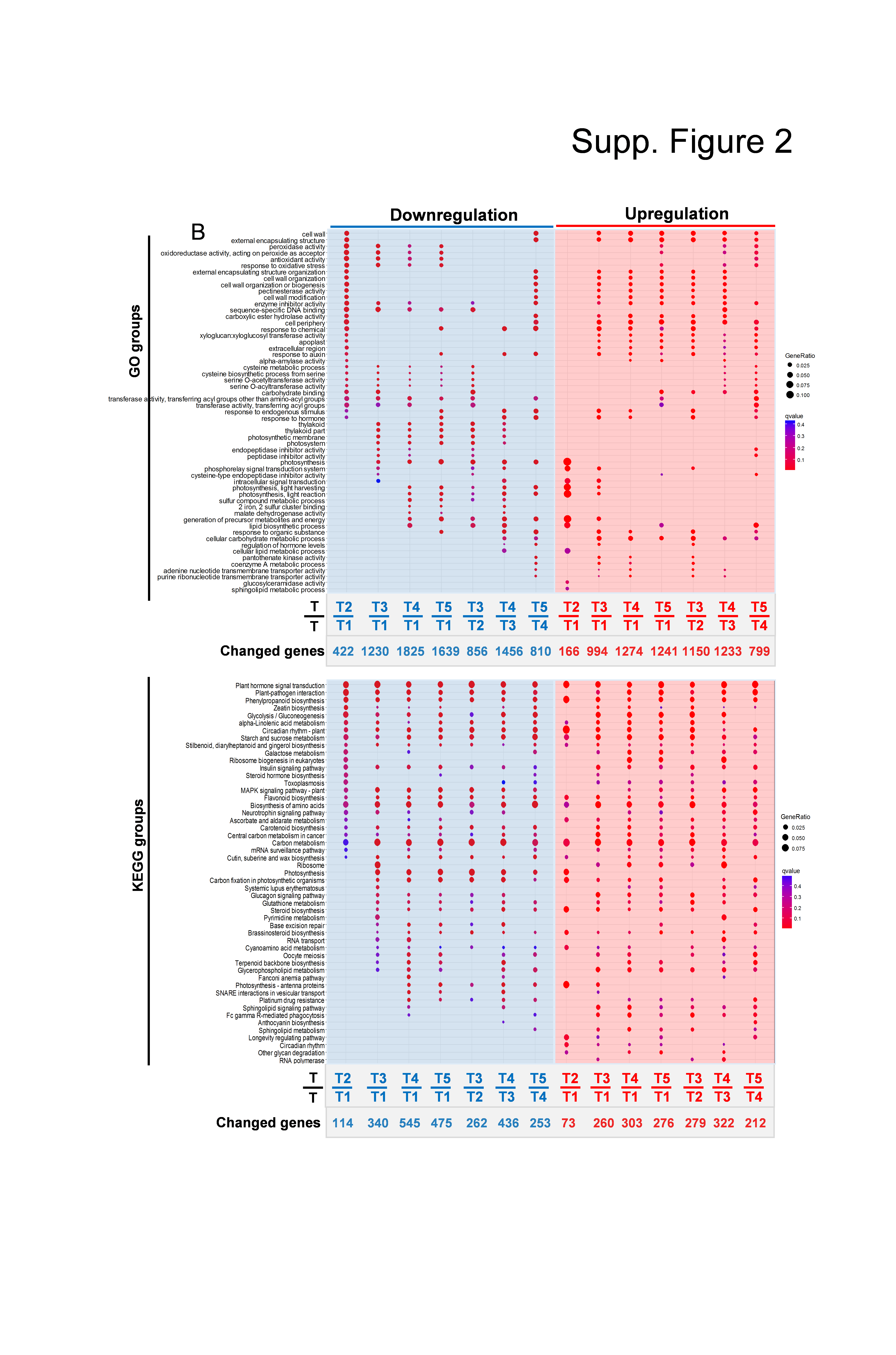

Supplement: Supplementary file 2 — Global transcriptome description of the flower opening and closure. Corresponding to Fig. 4. Global analysis of GO items and KEGG pathways during the flower opening and closure processes are shown. The transcriptome at 6:00 (T1) was used as the control, and the transcriptomes at 7:00 (T2), 10:00(T3), 14:00 (T4) and 18:00 (T5) were individually compared with each other (T/T comparisons). The total changed numbers are listed below. Red represents the upregulated groups, and blue marks the downregulated groups (TIF 1165 kb) [file 12870_2018_1357_MOESM2_ESM.tif]

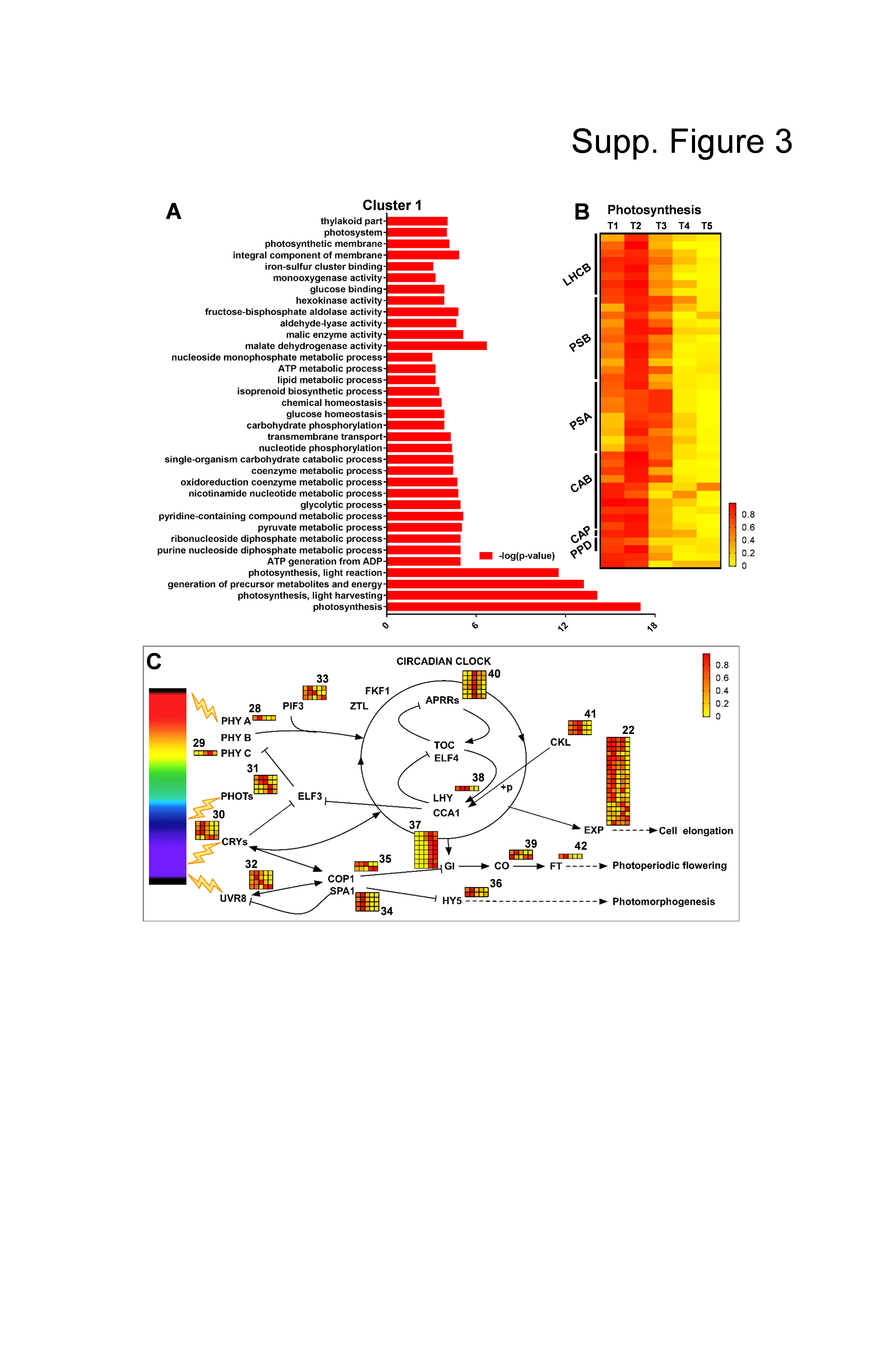

Supplement: Supplementary file 3 — The involvement of light signaling during flower opening. (A-B) GO items and heatmaps provided the transcriptome profiles of cluster 1, including genes involved in photosynthesis (list in Additional file 4: Table S1). LHCB, Chlorophyll a-b binding protein; PSB, Oxygen-evolving enhancer protein; PSA, Photosystem I reaction center subunit V; CAB, Chlorophyll a-b binding protein; CAP, Chlorophyll a-b binding protein CP24; PPD, PsbP domain-containing protein; (C) Transcript profiling of light signaling network. The basic red/far-red light signaling cascade is based on the PHYs-PIF signaling module, as referred in [87]. Blue light signaling primarily consists of the CRY-COP1/SPA1-HY5 signaling module for photomorphogenesis, CRY-COP1/SPA1-GI-CO-FT module for photoperiodic flowering, PHOTs-mediated signaling for phototropism, as referred to [8, 70, 73]. UVB-triggered signaling is established by UVR8-COP1/SPA1-HY5 module, as referred in [72]. The input light signals additionally stimulate the endogenous circadian oscillator, which is feedback regulated by the transcriptional loop of the APRRs-TOCCCA1-LHY-ELF4-FKF1-ZTL clock signaling module, as referred to [88]. PHY, Phytochrome; CRY, Cryptochrome; PHOT, Phototropin; UVR8, UV-B resistance 8; PIF3, Phytochrome-interacting factor 3; ELF, Early flowering; COP1, Constitutive photomorphogenic 1; SPA1, Suppressor of phytochrome A1; HY5, Elongated hypocotyl 5; FKF1, Flavin Binding, Kelch Repeat, F-BOX1; ZTL, ZEITLUPE; APRR, Pseudo-Response Regulators; TOC1, Timing of CAB expression1; LHY, Late Elongated Hypocotyl; CCA1, Circadian Clock Associated 1; GI, GIGANTEA; CO, CONSTANS; FT, Protein Flowering Locus T; CKL, Casein kinase 1-like; EXP, Expansin. The expression patterns of these genes are marked as heatmaps (the corresponding genes were listed in Additional file 4: Table S4 according to the labelled number “28” to “42” in each cluster). (TIF 2690 kb) [file 12870_2018_1357_MOESM3_ESM.tif]

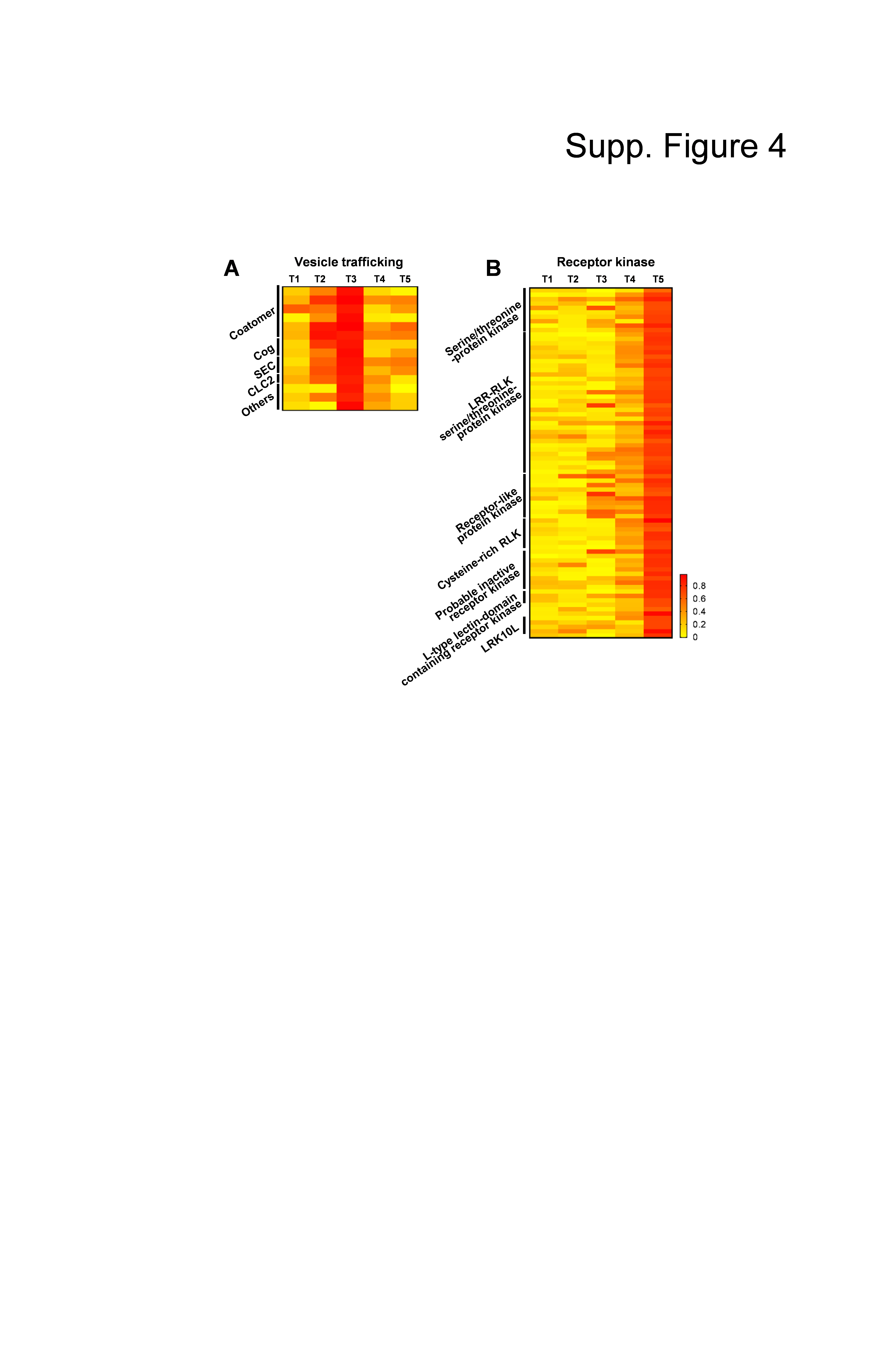

Supplement: Supplementary file 5 — Transcriptome description of vesicle trafficking and receptor kinase-mediated cascades. (A) Heatmap provided the transcriptome profiles of vesicle trafficking-related genes in cluster 3. The corresponding genes were listed in Additional file 4: Table S2. (B) Heatmap provided the transcriptome profiles of RLKs in cluster 8. The corresponding genes were listed in Additional file 4: Table S3. (TIF 640 kb) [file 12870_2018_1357_MOESM5_ESM.tif]

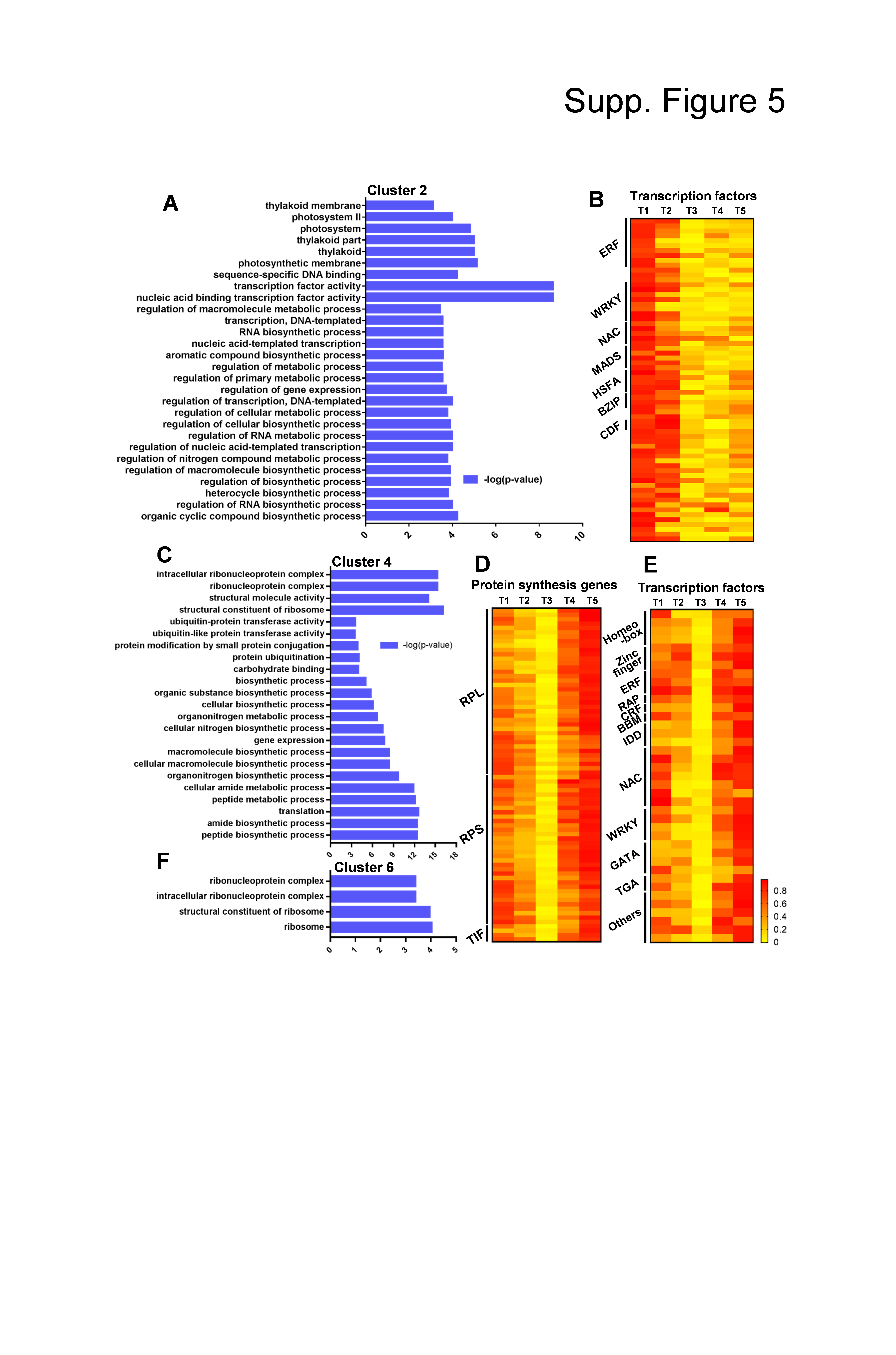

Supplement: Supplementary file 6 — Description of flower opening-associated signaling events with downregulations. (A-F) GO items and heatmaps provided the transcriptome profiles of cluster 2, 4 and 6, mainly including transcriptional factors and protein synthesis-related genes (list in Additional file 4: Tables S5-S7). In B, ERF, Ethylene-responsive transcription factor; WRKY, WRKY transcription factor; NAC, NAC domain-containing protein; MADS, MADS-box transcription factor; HSFA, Heat stress transcription factor; BZIP, Basic leucine zipper; CDF, Cyclic dof factor. In D, RPL, ribosomal protein L; RPS, ribosomal protein S; TIF, Eukaryotic translation initiation factor. In E, Homeo-box, Homeobox-leucine zipper protein; Zinc finger, Dof zinc finger protein; ERF, Ethylene-responsive transcription factor; RAP, Ethylene-responsive transcription factor RAP; CRF, Ethylene-responsive transcription factor CRF; BBM, AP2-like ethylene-responsive transcription factor BBM; IDD, indeterminate-domain; NAC, NAC domain-containing protein; WRKY, WRKY transcription factor; GATA, GATA transcription factor; TGA, Transcription factor TGA. (TIF 3371 kb) [file 12870_2018_1357_MOESM6_ESM.tif]
