# Supplementary material for: Auxin controls circadian flower opening and closure in the waterlily
Source: BMC Plant Biol. 2018 Jul 11;18:143. doi: 10.1186/s12870-018-1357-7 (PMC6042438; doi:10.1186/s12870-018-1357-7)
Supplement: Supplementary file 4 — Table S1. Transcriptome profile of photosynthesis-regulators, corresponding to Additional file 3: Figure S3B. Table S2. Transcriptome profile of vesicle trafficking-regulators, corresponding to Additional file 5: Figure S4A. Table S3. Transcriptome profile of receptor kinases, corresponding to Additional file 5: Figure S4B. Table S4. Transcriptome profile of light signaling-regulators, corresponding to Additional file 3: Figure S3C. Table S5. Transcriptome profile of transcriptional factors, corresponding to Additional file 6: Figure S5B. Table S6. Transcriptome profile of protein synthesis-related genes, corresponding to Additional file 6: Figure S5D. Table S7. Transcriptome profile of transcriptional factors, corresponding to Additional file 6: Figure S5E. Table S8. NGS reads for waterlily RNA. Table S9. Statistical result of waterlily RNA transcriptome assembly. (DOCX 65 kb) [file 12870_2018_1357_MOESM4_ESM.docx]

| Table S1 Photosynthesis (corresponding to Supp. Fig. 3B) | | | | | | |
| --- | --- | --- | --- | --- | --- | --- |
| Gene ID | **Short description** | **FPKM (6:00)** | **FPKM (7:00)** | **FPKM (10:00)** | **FPKM (14:00)** | **FPKM (18:00)** |
| DN31464_c0_g1 | Photosystem I reaction center subunit V | 5.1 | 11.2 | 5.0 | 2.9 | 1.9 |
| DN31464_c0_g2 | Photosystem I reaction center subunit V | 190.7 | 302.2 | 98.4 | 19.8 | 22.1 |
| DN36729_c0_g5 | Photosystem II 10 kDa polypeptide | 103.3 | 115.7 | 82.5 | 58.8 | 42.3 |
| DN47247_c2_g2 | Photosystem II 10 kDa polypeptide | 565.5 | 579.7 | 465.7 | 334.9 | 257.7 |
| DN41412_c0_g1 | Photosystem II reaction center W protein | 637.8 | 710.0 | 429.1 | 252.0 | 211.5 |
| DN30047_c0_g1 | Photosystem I reaction center subunit psaK | 436.1 | 525.2 | 256.4 | 51.6 | 62.0 |
| DN38927_c1_g1 | Photosystem I reaction center subunit VI-2 | 99.6 | 110.3 | 73.1 | 58.6 | 39.7 |
| DN39294_c3_g1 | Photosystem I reaction center subunit III | 649.2 | 745.1 | 388.8 | 189.5 | 184.1 |
| DN43070_c0_g1 | Photosystem I reaction center subunit IV A | 175.9 | 193.0 | 181.5 | 143.4 | 96.0 |
| DN34189_c1_g1 | PsbP domain-containing protein 3 | 11.7 | 16.7 | 15.0 | 11.1 | 9.5 |
| DN32070_c0_g1 | PsbP domain-containing protein 5 | 12.1 | 13.8 | 10.3 | 6.8 | 8.9 |
| DN46990_c2_g4 | Oxygen-evolving enhancer protein 1 | 6.2 | 10.3 | 8.0 | 3.6 | 3.2 |
| DN37701_c0_g1 | Photosynthetic NDH subunit of lumenal location 1 | 7.4 | 9.6 | 9.3 | 5.1 | 5.0 |
| DN29565_c0_g1 | Chlorophyll a-b binding protein CP24 10A | 582.8 | 781.0 | 468.6 | 101.1 | 35.9 |
| DN46141_c0_g1 | Chlorophyll a-b binding protein CP26 | 157.7 | 291.0 | 190.9 | 32.6 | 39.8 |
| DN44338_c3_g1 | Chlorophyll a-b binding protein CP26 | 116.1 | 196.3 | 111.3 | 18.9 | 23.3 |
| DN44338_c3_g2 | Chlorophyll a-b binding protein CP26 | 8.0 | 20.5 | 5.9 | 0.8 | 2.4 |
| DN44338_c3_g3 | Chlorophyll a-b binding protein CP26 | 490.0 | 796.6 | 612.7 | 107.1 | 148.3 |
| DN47103_c3_g2 | Chlorophyll a-b binding protein CP29.1 | 125.8 | 149.8 | 78.4 | 47.7 | 42.6 |
| DN29216_c0_g8 | Chlorophyll a-b binding protein CP29.2 | 341.8 | 443.3 | 261.7 | 136.6 | 119.8 |
| DN37612_c0_g1 | Chlorophyll a-b binding protein 36 | 596.3 | 707.1 | 798.5 | 94.1 | 31.4 |
| DN29098_c1_g2 | Chlorophyll a-b binding protein | 1077.6 | 1574.5 | 1740.8 | 185.3 | 48.6 |
| DN29098_c1_g1 | Chlorophyll a-b binding protein of LHCII type I | 1171.3 | 1580.7 | 1791.2 | 181.5 | 53.4 |
| DN33505_c0_g1 | Chlorophyll a-b binding protein 3C | 510.7 | 1458.0 | 1362.4 | 386.4 | 109.5 |
| DN29098_c1_g3 | Chlorophyll a-b binding protein 3C | 178.5 | 550.3 | 486.8 | 145.0 | 34.4 |
| DN30014_c0_g3 | Chlorophyll a-b binding protein 7 | 23.4 | 68.5 | 40.1 | 10.9 | 3.9 |
| DN31016_c2_g4 | Chlorophyll a-b binding protein 1D | 45.2 | 133.3 | 126.2 | 61.4 | 29.2 |
| DN30014_c0_g4 | Chlorophyll a-b binding protein 7 | 172.0 | 533.6 | 428.6 | 87.1 | 24.2 |
| DN44517_c1_g1 | Chlorophyll a-b binding protein 4 | 404.4 | 521.6 | 340.9 | 136.5 | 110.4 |
| DN39846_c1_g1 | Chlorophyll a-b binding protein 8 | 1010.4 | 1457.9 | 1175.1 | 148.8 | 114.5 |
| DN36707_c2_g1 | Chlorophyll a-b binding protein 7 | 110.0 | 129.6 | 53.9 | 14.2 | 11.5 |
| DN35755_c0_g1 | Chlorophyll a-b binding protein 6A | 347.2 | 542.2 | 453.7 | 157.5 | 125.1 |
| DN35911_c1_g1 | Oxygen-evolving enhancer protein 3-2 | 112.5 | 121.4 | 19.9 | 39.3 | 31.8 |
| DN29112_c0_g1 | Oxygen-evolving enhancer protein 3-1 | 86.2 | 80.1 | 45.4 | 40.3 | 62.9 |
| DN35911_c1_g6 | Oxygen-evolving enhancer protein 3-2 | 6.4 | 4.9 | 1.0 | 3.4 | 0.6 |
| DN37545_c1_g1 | Oxygen-evolving enhancer protein 2 | 566.8 | 559.5 | 323.6 | 269.6 | 202.3 |
| DN46990_c2_g2 | Oxygen-evolving enhancer protein 1 | 830.5 | 847.4 | 479.3 | 298.7 | 329.6 |
| DN36988_c2_g2 | Photosystem I reaction center subunit | 233.5 | 243.4 | 101.5 | 36.6 | 26.8 |
| DN40540_c0_g1 | Photosystem I reaction center subunit XI | 675.8 | 794.1 | 390.1 | 251.0 | 188.2 |
| DN34116_c0_g2 | Photosystem I reaction center subunit II | 264.5 | 270.1 | 211.0 | 205.3 | 171.9 |
| DN26923_c0_g1 | Photosystem II reaction center X protein | 317.5 | 294.2 | 183.1 | 184.2 | 175.7 |
| DN31926_c0_g1 | Photosystem II core complex proteins psbY | 288.9 | 349.5 | 137.5 | 62.7 | 85.0 |
| DN31628_c2_g1 | Cytochrome b6-f complex iron-sulfur subunit | 491.4 | 482.9 | 341.6 | 215.2 | 222.1 |
|  |  |  |  |  |  |  |

| Table S2 Vesicle trafficking (corresponding to Supp. Fig. 4A) | | | | | | |
| --- | --- | --- | --- | --- | --- | --- |
| Gene ID | **Best homologs** | **FPKM (6:00)** | **FPKM (7:00)** | **FPKM (10:00)** | **FPKM (14:00)** | **FPKM (18:00)** |
| DN43037_c0_g1 | Coatomer subunit gamma-2 | 121.9 | 135.8 | 156.7 | 119.8 | 114.2 |
| DN31342_c0_g1 | Coatomer subunit beta-1 | 32.1 | 43.2 | 47.6 | 35.3 | 36.5 |
| DN43414_c0_g2 | Coatomer subunit beta-1 | 48.3 | 46.9 | 52.7 | 40.1 | 44.4 |
| DN46706_c0_g1 | Coatomer subunit beta-1 | 35.7 | 42.7 | 52.2 | 36.4 | 35.8 |
| DN46706_c0_g3 | Coatomer subunit beta-1 | 25.0 | 33.2 | 34.5 | 26.7 | 29.4 |
| DN47285_c3_g2 | Coatomer subunit beta-1 | 144.9 | 190.9 | 187.4 | 158.8 | 160.7 |
| DN47538_c2_g1 | Cog8 | 24.4 | 30.7 | 32.0 | 24.5 | 24.6 |
| DN41955_c0_g1 | Cog5 | 15.3 | 19.0 | 23.8 | 14.9 | 17.4 |
| DN45756_c1_g1 | SEC24 | 58.6 | 73.7 | 82.9 | 69.0 | 70.8 |
| DN45680_c1_g1 | SEC23 | 25.0 | 34.2 | 39.0 | 26.0 | 29.5 |
| DN36018_c0_g1 | CLC2 | 135.4 | 148.8 | 159.6 | 141.0 | 126.4 |
| DN25492_c0_g1 | GNT2 | 15.4 | 14.7 | 30.9 | 19.7 | 13.7 |
| DN38966_c0_g1 | AP-1 complex subunit gamma-2 | 32.1 | 36.5 | 40.7 | 35.3 | 32.0 |
| DN44207_c0_g1 | DNA damage-binding protein 2 | 40.0 | 30.8 | 112.9 | 58.9 | 45.2 |

| Table S3 Receptor Kinases (corresponding to Supp. Fig. 4B) | | | | | | |
| --- | --- | --- | --- | --- | --- | --- |
| Gene ID | **Best homologs** | **FPKM (6:00)** | **FPKM (7:00)** | **FPKM (10:00)** | **FPKM (14:00)** | **FPKM (18:00)** |
| DN33339_c0_g1 | BSK3 | 15.6 | 15.2 | 5.1 | 11.8 | 42.7 |
| DN37940_c0_g1 | NEK5 | 2.7 | 3.5 | 3.2 | 8.3 | 13.4 |
| DN37943_c0_g1 | AFC1 | 7.9 | 9.4 | 8.7 | 10.4 | 12.0 |
| DN34114_c2_g1 | D6PKL2 | 10.3 | 11.8 | 9.2 | 12.9 | 17.1 |
| DN38594_c0_g2 | HT1 | 3.4 | 2.5 | 4.3 | 3.0 | 4.4 |
| DN44295_c0_g1 | PBL7 | 5.0 | 5.8 | 5.7 | 7.9 | 12.2 |
| DN32686_c0_g1 | At1g01540 | 6.5 | 5.5 | 6.9 | 13.3 | 19.2 |
| DN38326_c0_g1 | STY46 | 38.1 | 32.3 | 38.2 | 32.2 | 46.2 |
| DN33303_c0_g1 | PBL26 | 8.6 | 9.9 | 14.7 | 20.6 | 24.7 |
| DN47420_c2_g1 | At1g01540 | 12.2 | 9.1 | 11.5 | 20.2 | 27.2 |
| DN47328_c0_g1 | At1g53440 | 4.8 | 3.9 | 3.8 | 13.4 | 24.3 |
| DN31555_c1_g1 | At3g47570 | 0.5 | 0.7 | 0.4 | 1.5 | 4.2 |
| DN43158_c0_g1 | At1g51890 | 10.9 | 9.5 | 11.1 | 18.7 | 30.3 |
| DN47347_c3_g1 | At1g56140 | 59.5 | 48.3 | 48.8 | 77.3 | 101.7 |
| DN33044_c0_g1 | At1g56130 | 36.7 | 35.3 | 31.1 | 51.6 | 70.4 |
| DN37919_c0_g2 | RFK1 | 10.9 | 11.5 | 10.1 | 12.7 | 14.7 |
| DN31395_c3_g1 | At4g37250 | 1.1 | 1.1 | 1.4 | 3.3 | 6.1 |
| DN46412_c1_g1 | At1g56140 | 23.7 | 30.4 | 22.3 | 45.5 | 60.2 |
| DN41824_c0_g1 | IRK | 0.7 | 1.7 | 0.6 | 1.6 | 4.4 |
| DN38653_c2_g2 | At1g34110 | 1.6 | 1.6 | 1.2 | 1.4 | 2.7 |
| DN44168_c0_g1 | At1g12460 | 2.6 | 2.9 | 5.0 | 5.8 | 11.1 |
| DN42800_c1_g1 | IRK | 2.6 | 2.8 | 1.5 | 3.6 | 7.9 |
| DN46904_c0_g1 | At1g53430 | 2.8 | 2.7 | 2.2 | 8.5 | 16.4 |
| DN42213_c1_g1 | RCH1 | 0.5 | 0.6 | 1.5 | 1.4 | 2.0 |
| DN42819_c1_g2 | RGI3 | 3.3 | 3.0 | 1.1 | 7.2 | 18.5 |
| DN42819_c1_g1 | RGI3 | 11.1 | 15.4 | 14.4 | 18.9 | 28.4 |
| DN43959_c2_g1 | RPK2 | 2.8 | 3.7 | 8.9 | 4.6 | 8.0 |
| DN28900_c2_g2 | IOS1 | 2.3 | 1.7 | 1.2 | 1.6 | 4.6 |
| DN40823_c2_g2 | IOS1 | 7.2 | 6.3 | 8.0 | 15.8 | 23.3 |
| DN44118_c1_g2 | IOS1 | 2.9 | 2.9 | 2.2 | 2.2 | 5.2 |
| DN47537_c2_g3 | RGI3 | 7.7 | 13.9 | 13.3 | 16.3 | 23.8 |
| DN46995_c0_g1 | RGI3 | 1.7 | 2.0 | 0.8 | 4.9 | 11.6 |
| DN44715_c1_g1 | At2g19230 | 2.9 | 2.6 | 2.0 | 2.9 | 6.4 |
| DN28879_c1_g1 | At2g19230 | 3.8 | 4.4 | 3.3 | 3.9 | 5.6 |
| DN28879_c0_g1 | At2g04300 | 0.8 | 1.0 | 0.5 | 1.1 | 2.3 |
| DN45847_c0_g1 | At5g63930 | 4.0 | 4.3 | 4.5 | 9.3 | 14.2 |
| DN45223_c0_g1 | LRR-RLK | 19.6 | 21.0 | 26.8 | 35.9 | 42.9 |
| DN36693_c4_g2 | BAM1 | 8.8 | 7.2 | 13.0 | 13.0 | 16.4 |
| DN47319_c1_g1 | BAM1 | 15.4 | 16.2 | 29.4 | 27.7 | 39.3 |
| DN38535_c0_g1 | BAM2 | 3.3 | 3.2 | 3.7 | 5.6 | 7.4 |
| DN44490_c1_g1 | PXC3 | 23.0 | 26.5 | 22.0 | 32.6 | 48.3 |
| DN31057_c2_g1 | BAM1 | 15.7 | 16.5 | 25.5 | 27.5 | 37.6 |
| DN30812_c1_g1 | At4g10390 | 83.1 | 102.0 | 105.4 | 91.9 | 104.7 |
| DN46220_c2_g2 | HSL1 | 22.9 | 19.9 | 26.1 | 62.4 | 84.5 |
| DN43119_c1_g1 | THE1 | 4.0 | 3.9 | 11.9 | 7.7 | 14.8 |
| DN21680_c0_g1 | FERONIA | 0.3 | 0.3 | 0.2 | 0.3 | 0.7 |
| DN40760_c0_g1 | FERONIA | 35.3 | 33.4 | 93.9 | 48.0 | 82.3 |
| DN31506_c0_g1 | HAIKU2 | 4.6 | 4.0 | 5.2 | 5.8 | 6.4 |
| DN43081_c1_g2 | RLP51 | 2.2 | 2.4 | 8.1 | 8.5 | 14.5 |
| DN29160_c2_g2 | TMK1 | 46.5 | 48.4 | 59.5 | 57.6 | 78.1 |
| DN37767_c0_g1 | TMK1 | 24.2 | 31.0 | 43.0 | 39.3 | 49.1 |
| DN37617_c0_g1 | TMK4 | 0.1 | 0.1 | 1.5 | 0.5 | 1.8 |
| DN31057_c3_g2 | CRK10 | 2.2 | 1.4 | 1.5 | 3.5 | 5.8 |
| DN34131_c2_g1 | CRK10 | 10.7 | 8.7 | 9.1 | 19.8 | 25.6 |
| DN37899_c1_g1 | CRK25 | 1.8 | 1.4 | 1.2 | 2.9 | 4.4 |
| DN30086_c0_g1 | CRRSP38 | 6.7 | 5.8 | 5.8 | 8.8 | 14.8 |
| DN30535_c0_g1 | At2g26730 | 7.1 | 6.7 | 8.7 | 13.6 | 21.5 |
| DN35079_c0_g1 | At5g58300 | 1.3 | 1.8 | 0.9 | 4.3 | 7.4 |
| DN39160_c1_g1 | At5g67200 | 1.8 | 1.4 | 1.3 | 4.4 | 9.9 |
| DN46809_c1_g3 | RKL1 | 12.1 | 12.0 | 26.2 | 21.7 | 29.0 |
| DN46809_c1_g1 | RKL1 | 1.3 | 1.6 | 3.0 | 2.9 | 8.4 |
| DN41132_c0_g2 | At5g58300 | 2.7 | 2.7 | 1.6 | 4.6 | 9.0 |
| DN45746_c0_g1 | At3g03770 | 6.5 | 9.9 | 4.2 | 7.4 | 14.1 |
| DN46505_c0_g2 | At5g48380 | 42.2 | 37.0 | 33.2 | 62.5 | 89.6 |
| DN37148_c0_g1 | At3g03770 | 2.1 | 1.3 | 1.1 | 3.1 | 12.7 |
| DN31563_c0_g1 | LYK3 | 2.1 | 1.3 | 2.2 | 2.9 | 4.0 |
| DN34566_c0_g1 | LYK4 | 0.7 | 0.6 | 0.6 | 1.0 | 1.3 |
| DN35740_c0_g1 | LYM2 | 8.1 | 8.3 | 5.7 | 8.0 | 15.5 |
| DN35419_c1_g1 | LECRK91 | 1.3 | 1.4 | 1.0 | 1.5 | 5.5 |
| DN43957_c0_g2 | LECRKS1 | 1.3 | 1.0 | 1.4 | 1.7 | 2.1 |
| DN28504_c0_g2 | LECRK82 | 1.4 | 1.1 | 1.0 | 1.4 | 2.7 |
| DN47894_c0_g1 | RKS1 | 2.9 | 2.5 | 3.5 | 4.2 | 8.0 |
| DN42490_c1_g2 | At2g19130 | 8.5 | 10.9 | 8.3 | 10.3 | 13.6 |
| DN35822_c0_g1 | At1g52310 | 50.2 | 54.9 | 57.4 | 67.7 | 97.8 |
| DN29528_c0_g1 | LRK10L-1.5 | 1.7 | 1.7 | 1.5 | 3.9 | 5.5 |
| DN34659_c0_g1 | LRK10L-1.2 | 44.7 | 42.1 | 45.2 | 40.4 | 55.9 |
| DN36390_c2_g1 | LRK10L-1.2 | 15.4 | 18.9 | 19.9 | 16.9 | 26.3 |
| DN43876_c0_g2 | LRK10L-1.2 | 9.7 | 11.8 | 7.6 | 8.8 | 16.4 |
| DN31912_c0_g1 | MKK7 | 46.7 | 39.8 | 20.4 | 50.3 | 116.6 |
| DN34158_c0_g1 | MAPKKK18 | 3.0 | 1.6 | 2.2 | 11.7 | 17.2 |
| DN33995_c1_g1 | RPPL1 | 9.9 | 12.9 | 14.5 | 19.1 | 25.0 |
| DN33071_c2_g2 | RPP13L4 | 17.3 | 20.5 | 26.8 | 29.4 | 48.3 |
| DN30877_c1_g1 | TAO1 | 4.2 | 3.8 | 4.6 | 5.3 | 7.0 |
| DN33553_c3_g1 | RPP13L4 | 17.8 | 22.7 | 17.3 | 19.6 | 32.9 |
| DN47179_c1_g1 | RGA2 | 5.2 | 6.1 | 4.9 | 4.9 | 7.5 |
| DN36246_c3_g2 | TAO1 | 61.5 | 84.2 | 63.7 | 70.7 | 109.1 |
| DN32866_c1_g4 | PRR1 | 0.4 | 0.3 | 0.7 | 0.7 | 1.3 |
| DN30588_c0_g1 | TAO1 | 2.9 | 2.4 | 2.3 | 2.4 | 4.6 |
| DN30236_c1_g1 | RPPL1 | 59.5 | 77.1 | 61.4 | 58.7 | 102.4 |
| DN36294_c0_g1 | RGA1 | 30.5 | 38.5 | 33.8 | 32.5 | 58.5 |
| DN33995_c1_g3 | At3g14460 | 23.1 | 20.7 | 24.9 | 38.7 | 68.2 |
| DN36657_c1_g3 | RE2 | 0.2 | 0.3 | 0.4 | 0.5 | 1.2 |
| DN29880_c0_g1 | RE1 | 0.2 | 0.4 | 0.3 | 0.7 | 0.8 |
| DN47035_c2_g7 | POL | 9.4 | 10.8 | 6.5 | 13.3 | 21.1 |
| DN41792_c6_g2 | RE1 | 0.7 | 0.4 | 0.8 | 0.9 | 2.5 |
| DN35213_c0_g1 | inlI | 42.3 | 78.3 | 79.3 | 141.6 | 193.5 |
| DN30865_c1_g1 | inlI | 20.2 | 22.2 | 30.2 | 44.4 | 87.3 |
| DN33167_c0_g1 | MTP12 | 25.2 | 27.9 | 24.1 | 27.4 | 31.0 |
| DN39991_c1_g1 | CERK1 | 73.1 | 80.6 | 67.3 | 70.5 | 92.5 |
| DN43465_c2_g1 | USP39 | 9.9 | 9.3 | 9.8 | 11.0 | 13.3 |
| DN42399_c2_g1 | PUB26 | 18.7 | 16.7 | 29.5 | 31.4 | 69.8 |
| DN46926_c2_g2 | At5g61370 | 9.0 | 12.2 | 9.3 | 11.9 | 14.8 |
| DN41004_c0_g1 | KOR2 | 0.6 | 1.2 | 2.7 | 6.1 | 8.6 |
| DN30963_c1_g1 | PTI1-like | 80.2 | 80.1 | 77.5 | 98.1 | 109.7 |
| DN46176_c1_g1 | YLS3 | 205.0 | 219.3 | 230.8 | 338.3 | 474.0 |
| DN38653_c2_g1 | PERK8 | 1.1 | 1.2 | 0.4 | 1.5 | 3.7 |
| DN36546_c0_g1 | At5g03700 | 0.2 | 0.1 | 0.2 | 1.3 | 2.9 |
| DN33864_c1_g1 | DTX35 | 0.9 | 2.1 | 2.0 | 2.6 | 3.1 |
| DN35287_c0_g1 | SUPPRESSOR/ npr1-1 | 14.3 | 8.9 | 10.8 | 16.7 | 27.3 |
| DN44147_c1_g2 | ATL21B | 6.4 | 8.1 | 5.6 | 6.5 | 16.3 |
| DN33957_c0_g1 | ATL46 | 2.5 | 2.4 | 2.2 | 2.6 | 4.5 |
| DN35902_c0_g2 | TBL34 | 2.2 | 1.5 | 2.1 | 4.5 | 8.7 |
| DN47180_c1_g1 | TMV resistance protein N | 22.1 | 29.1 | 30.7 | 38.1 | 43.9 |
| DN46207_c0_g1 | alr3466 | 22.9 | 25.8 | 22.7 | 26.8 | 32.7 |

| **Table S4 Light signaling (corresponding to Supp. Fig. 3C)** | | | | | | | |
| --- | --- | --- | --- | --- | --- | --- | --- |
| **Heat Map**  **Number** | **Gene ID** | **Short Description** | **FPKM**  **(6:00)** | **FPKM**  **(7:00)** | **FPKM**  **(10:00)** | **FPKM**  **(14:00)** | **FPKM**  **(18:00)** |
| **28** | DN45078_c0_g1 | Phytochrome A | 54.2 | 46.8 | 136.0 | 196.6 | 95.1 |
| **29** | DN36376_c1_g1 | Phytochrome C | 18.1 | 23.9 | 14.8 | 11.9 | 14.8 |
| **30** | DN46663_c2_g3 | Cryptochrome DASH, chloroplastic/mitochondrial | 8.7 | 18.7 | 11.2 | 2.4 | 2.3 |
|  | DN46663_c2_g1 | Cryptochrome DASH, chloroplastic/mitochondrial | 10.7 | 18.5 | 9.3 | 3.7 | 1.9 |
|  | DN36206_c0_g1 | Cryptochrome-1 | 79.1 | 87.1 | 59.7 | 46.0 | 58.5 |
|  | DN38506_c1_g1 | Cryptochrome-2 | 26.6 | 23.0 | 34.1 | 37.7 | 32.0 |
| **31** | DN45604_c1_g1 | Phototropin-1 | 70.6 | 53.1 | 149.2 | 315.9 | 133.7 |
|  | DN45604_c1_g3 | Phototropin-1 | 10.7 | 7.4 | 21.5 | 47.9 | 21.1 |
|  | DN40426_c2_g1 | Phototropin-2 | 22.2 | 35.8 | 36.0 | 12.8 | 9.8 |
|  | DN47360_c1_g1 | Phototropin-2 | 18.8 | 34.3 | 38.6 | 12.2 | 7.9 |
| **32** | DN41296_c0_g1 | Ultraviolet-B receptor UVR8 | 16.3 | 18.2 | 15.1 | 11.5 | 11.3 |
|  | DN38788_c0_g1 | Ultraviolet-B receptor UVR8 | 11.5 | 34.3 | 10.4 | 1.6 | 5.3 |
|  | DN34302_c1_g1 | Ultraviolet-B receptor UVR8 | 20.1 | 25.1 | 39.1 | 13.7 | 6.2 |
|  | DN42939_c3_g1 | Ultraviolet-B receptor UVR8 | 49.2 | 49.6 | 38.8 | 55.0 | 56.3 |
| **33** | DN38877_c2_g1 | Transcription factor PIF1 | 7.3 | 13.3 | 5.2 | 4.1 | 7.9 |
|  | DN40120_c0_g1 | Transcription factor PIF1 | 4.0 | 6.5 | 7.6 | 0.5 | 0.5 |
|  | DN34043_c0_g1 | Transcription factor PIF5 | 64.7 | 61.4 | 50.0 | 63.3 | 75.1 |
| **34** | DN38330_c1_g2 | Protein SPA1-RELATED 3 | 61.4 | 90.8 | 31.4 | 7.4 | 10.1 |
|  | DN38330_c1_g1 | Protein SPA1-RELATED 3 | 110.0 | 196.8 | 74.6 | 23.8 | 38.3 |
|  | DN47846_c4_g2 | Protein SPA1-RELATED 3 | 120.6 | 159.3 | 70.9 | 22.2 | 35.9 |
|  | DN47846_c4_g1 | Protein SPA1-RELATED 3 | 39.0 | 62.8 | 25.2 | 4.3 | 9.1 |
| **35** | DN36413_c0_g1 | E3 ubiquitin-protein ligase COP1 | 19.5 | 19.3 | 21.2 | 14.4 | 14.6 |
|  | DN38313_c2_g1 | Constitutive photomorphogenesis protein 10 COP10 | 19.8 | 18.6 | 14.8 | 30.0 | 32.1 |
| **36** | DN29709_c0_g1 | Transcription factor HY5 | 23.0 | 30.2 | 18.5 | 18.9 | 15.9 |
|  | DN34255_c0_g1 | Transcription factor HY5-like | 102.6 | 103.9 | 67.6 | 57.0 | 75.5 |
| **37** | DN38848_c1_g1 | Protein GIGANTEA | 21.3 | 17.7 | 171.8 | 288.0 | 189.8 |
|  | DN46831_c2_g2 | Protein GIGANTEA | 26.2 | 26.3 | 247.9 | 418.5 | 296.3 |
|  | DN46831_c3_g2 | Protein GIGANTEA | 4.2 | 3.0 | 5.8 | 22.5 | 26.0 |
|  | DN26589_c0_g1 | Protein GIGANTEA | 8.1 | 5.0 | 7.4 | 37.3 | 44.9 |
|  | DN41575_c0_g1 | Protein GIGANTEA | 4.2 | 4.2 | 6.5 | 31.3 | 37.5 |
|  | DN38848_c0_g1 | Protein GIGANTEA | 4.6 | 4.2 | 5.8 | 31.8 | 38.5 |
|  | DN38848_c1_g3 | Protein GIGANTEA | 0.7 | 1.4 | 2.4 | 11.0 | 9.2 |
|  | DN46831_c3_g3 | Protein GIGANTEA | 6.4 | 5.8 | 7.7 | 37.4 | 42.3 |
| **38** | DN47504_c2_g1 | Protein LHY | 307.3 | 433.6 | 431.2 | 104.3 | 26.7 |
| **39** | DN47275_c1_g1 | Zinc finger protein CO3 | 216.7 | 349.5 | 183.9 | 41.4 | 74.6 |
|  | DN35415_c3_g1 | Zinc finger protein CO3 | 23.4 | 13.4 | 9.9 | 19.3 | 20.6 |
| **40** | DN48003_c3_g1 | Two-component response regulator-like PRR73 | 208.8 | 255.9 | 707.6 | 439.4 | 371.0 |
|  | DN37606_c1_g2 | Two-component response regulator-like APRR3 | 10.0 | 18.6 | 41.3 | 21.4 | 21.8 |
|  | DN48023_c2_g4 | Two-component response regulator-like PRR95 | 81.3 | 91.7 | 123.1 | 91.5 | 69.6 |
|  | DN38290_c1_g1 | Two-component response regulator-like APRR5 | 71.7 | 86.8 | 109.3 | 83.6 | 60.7 |
|  | DN47558_c1_g1 | Two-component response regulator-like PRR95 | 59.6 | 142.8 | 630.0 | 121.3 | 29.7 |
|  | DN37606_c1_g3 | Two-component response regulator-like PRR73 | 234.8 | 291.2 | 892.2 | 556.9 | 449.4 |
| **41** | DN46068_c0_g1 | Casein kinase 1-like protein 2 | 182.9 | 192.5 | 197.2 | 170.8 | 164.0 |
|  | DN33391_c2_g3 | Casein kinase 1-like protein 1 | 116.4 | 126.4 | 137.8 | 103.6 | 97.2 |
|  | DN29682_c0_g1 | Casein kinase 1-like protein 2 | 99.6 | 99.3 | 110.4 | 82.9 | 77.8 |
| **42** | DN34908_c2_g2 | Protein FLOWERING LOCUS T | 18.8 | 24.8 | 13.1 | 9.1 | 10.6 |

| Table S5 Transcriptional factor (corresponding to Supp. Fig. 5B) | | | | | | |
| --- | --- | --- | --- | --- | --- | --- |
| Gene ID | **Short description** | **FPKM (6:00)** | **FPKM (7:00)** | **FPKM (10:00)** | **FPKM (14:00)** | **FPKM (18:00)** |
| DN39804_c0_g1 | Ethylene-responsive transcription factor 3 | 190.7 | 198.6 | 63.8 | 82.7 | 94.2 |
| DN28940_c2_g2 | Ethylene-responsive transcription factor 9 | 66.6 | 53.3 | 16.3 | 20.7 | 24.1 |
| DN41274_c0_g1 | Ethylene-responsive transcription factor ERF105 | 37.8 | 26.0 | 11.7 | 17.2 | 15.4 |
| DN41274_c0_g2 | Pathogenesis-related genes transcriptional activator PTI5 | 2.7 | 2.1 | 0.2 | 0.1 | 0.2 |
| DN33328_c0_g1 | Ethylene-responsive transcription factor 9 | 107.5 | 73.7 | 31.3 | 72.5 | 42.4 |
| DN26579_c0_g1 | Ethylene-responsive transcription factor ERF113 | 2.2 | 0.2 | 0.0 | 0.5 | 0.2 |
| DN35982_c1_g1 | Ethylene-responsive transcription factor RAP2-13 | 16.2 | 7.9 | 6.2 | 6.3 | 6.7 |
| DN40525_c0_g1 | Ethylene-responsive transcription factor 4 | 28.3 | 22.7 | 6.4 | 14.3 | 11.9 |
| DN41736_c0_g1 | Ethylene-responsive transcription factor WRI1 | 15.7 | 16.2 | 13.2 | 10.4 | 13.1 |
| DN42015_c1_g5 | Ethylene-responsive transcription factor ERF017 | 8.0 | 2.0 | 0.2 | 1.6 | 0.5 |
| DN30091_c1_g1 | Ethylene-responsive transcription factor ERF114 | 17.4 | 9.7 | 2.9 | 4.5 | 2.6 |
| DN39252_c1_g1 | Auxin response factor 7 | 17.4 | 17.4 | 13.1 | 12.5 | 15.1 |
| DN43322_c1_g2 | Auxin-responsive protein IAA17 | 120.4 | 91.4 | 47.9 | 26.1 | 35.8 |
| DN29291_c6_g2 | Auxin-responsive protein IAA16 | 1378.1 | 1155.9 | 966.0 | 1013.8 | 880.1 |
| DN29735_c0_g1 | Probable WRKY transcription factor 28 | 13.9 | 12.3 | 7.9 | 5.0 | 8.7 |
| DN40440_c1_g1 | Probable WRKY transcription factor 40 | 4.6 | 4.0 | 1.1 | 1.0 | 1.4 |
| DN40440_c1_g2 | Probable WRKY transcription factor 40 | 5.2 | 1.9 | 0.8 | 1.9 | 1.1 |
| DN29768_c1_g1 | Probable WRKY transcription factor 28 | 8.3 | 7.3 | 4.3 | 3.8 | 4.1 |
| DN32281_c0_g1 | Probable WRKY transcription factor 41 | 9.4 | 2.4 | 2.7 | 2.3 | 2.9 |
| DN41988_c2_g2 | Probable WRKY transcription factor 11 | 10.3 | 4.3 | 4.6 | 3.5 | 4.9 |
| DN41388_c0_g3 | WRKY transcription factor 22 | 2.6 | 2.0 | 0.6 | 0.6 | 0.6 |
| DN30923_c0_g1 | WRKY transcription factor 22 | 1.2 | 0.9 | 0.3 | 0.3 | 0.5 |
| DN39956_c3_g2 | NAC domain-containing protein 43 | 2.7 | 1.7 | 0.9 | 0.8 | 1.3 |
| DN40107_c3_g1 | NAC domain-containing protein 100 | 5.9 | 3.9 | 2.7 | 3.2 | 4.1 |
| DN34736_c0_g2 | NAC domain-containing protein 78 | 22.0 | 19.3 | 17.8 | 16.1 | 17.8 |
| DN36165_c1_g1 | NAC transcription factor ONAC010 | 112.9 | 93.6 | 83.9 | 79.2 | 37.8 |
| DN47620_c1_g3 | NAC domain-containing protein 2 | 159.6 | 140.6 | 71.6 | 109.0 | 109.7 |
| DN35744_c1_g1 | MADS-box protein SVP | 18.6 | 11.9 | 9.9 | 10.9 | 9.4 |
| DN32394_c4_g1 | MADS-box transcription factor 6 | 12.1 | 15.3 | 7.7 | 6.4 | 7.3 |
| DN34161_c1_g2 | MADS-box protein SOC1 | 25.8 | 16.1 | 14.8 | 12.8 | 11.5 |
| DN47271_c0_g1 | MADS-box transcription factor 50 | 4.6 | 4.8 | 2.0 | 0.9 | 1.7 |
| DN40573_c2_g1 | Agamous-like MADS-box protein AGL21 | 56.9 | 47.4 | 43.0 | 36.5 | 37.3 |
| DN39352_c1_g2 | Heat stress transcription factor A-5 | 53.1 | 48.0 | 33.1 | 36.3 | 42.0 |
| DN30978_c0_g1 | Heat stress transcription factor A-2e | 11.0 | 10.6 | 7.8 | 4.4 | 8.9 |
| DN37913_c1_g1 | Heat stress transcription factor B-2a | 11.2 | 11.7 | 5.6 | 5.8 | 8.2 |
| DN45293_c1_g1 | Heat shock factor protein HSF24 | 126.5 | 128.2 | 74.4 | 85.0 | 106.5 |
| DN34870_c0_g1 | Heat stress transcription factor A-5 | 546.1 | 473.5 | 456.3 | 335.2 | 350.7 |
| DN39332_c0_g3 | Basic leucine zipper 43 | 10.3 | 9.4 | 6.7 | 7.2 | 7.5 |
| DN45252_c1_g4 | Basic leucine zipper 34 | 94.8 | 92.7 | 57.9 | 61.8 | 66.2 |
| DN42663_c2_g2 | Basic leucine zipper 43 | 24.4 | 18.8 | 8.3 | 12.1 | 16.7 |
| DN35130_c0_g1 | Homeobox-leucine zipper protein HOX6 | 59.0 | 39.6 | 22.8 | 29.5 | 40.7 |
| DN31765_c2_g1 | Homeobox-leucine zipper protein ATHB-40 | 28.2 | 33.1 | 16.2 | 21.6 | 19.8 |
| DN34057_c3_g1 | Cyclic dof factor 2 | 262.4 | 313.7 | 93.0 | 39.9 | 94.2 |
| DN36410_c1_g2 | Cyclic dof factor 2 | 309.1 | 352.0 | 102.8 | 44.3 | 101.0 |
| DN46008_c3_g1 | Zinc finger BED domain-containing protein RICESLEEPER 2 | 69.0 | 66.4 | 50.9 | 50.6 | 56.4 |
| DN38515_c1_g1 | Transcription factor E2FA | 20.6 | 20.1 | 14.5 | 11.9 | 14.6 |
| DN36241_c0_g1 | Trihelix transcription factor GT-4 | 23.3 | 21.7 | 18.4 | 16.6 | 20.9 |
| DN34255_c0_g1 | Transcription factor HY5-like | 102.6 | 103.9 | 67.6 | 57.0 | 75.5 |
| DN38867_c0_g2 | Paired amphipathic helix protein Sin3-like 4 | 3.6 | 1.4 | 0.9 | 1.4 | 1.9 |
| DN35012_c1_g1 | Protein MRG1 | 136.5 | 137.7 | 112.7 | 116.3 | 124.0 |
| DN31043_c2_g2 | High mobility group B protein 1 | 128.8 | 117.1 | 102.6 | 78.9 | 76.9 |
| DN41762_c1_g2 | Chaperone protein dnaJ 2 | 505.5 | 484.1 | 231.4 | 229.0 | 306.2 |
| DN33546_c3_g1 | Monothiol glutaredoxin-S17 | 37.0 | 34.2 | 31.7 | 26.9 | 28.2 |
| DN44727_c0_g1 | G-box-binding factor 3 | 93.9 | 123.8 | 71.5 | 87.6 | 91.1 |
| DN45383_c1_g2 | Transcription initiation factor IIF subunit alpha | 44.1 | 41.9 | 38.3 | 40.1 | 37.9 |
| DN42568_c1_g8 | RE1 | 3.4 | 4.5 | 0.7 | 2.5 | 2.3 |
| DN26931_c0_g1 | Ricin B-like lectin R40G3 | 82.4 | 77.6 | 54.7 | 42.9 | 63.9 |
| DN42054_c0_g1 | Homeotic protein knotted-1 | 80.8 | 53.2 | 64.6 | 66.9 | 46.6 |
| DN40480_c0_g1 | BEL1-like homeodomain protein 1 | 345.7 | 344.4 | 303.9 | 365.7 | 313.6 |
| DN36634_c0_g1 | RNA polymerase II transcriptional coactivator KIWI | 13.6 | 7.8 | 8.3 | 7.1 | 9.6 |
| DN41776_c5_g1 | Dof zinc finger protein DOF1.3 | 10.0 | 9.8 | 1.6 | 0.4 | 1.2 |
| DN36371_c3_g1 | Homeobox protein knotted-1-like LET6 | 14.9 | 8.5 | 8.7 | 8.3 | 8.5 |
| DN40419_c1_g1 | Ricin B-like lectin R40G3 | 82.5 | 64.8 | 65.4 | 75.4 | 67.8 |
| DN40431_c1_g2 | PHD finger protein ALFIN-LIKE 5 | 12.8 | 16.5 | 10.2 | 8.3 | 12.1 |
| DN43070_c1_g1 | PHD finger protein ING1 | 49.2 | 45.5 | 21.1 | 27.5 | 28.1 |

| Table S6 Protein synthesis genes (corresponding to Supp. Fig. 5D) | | | | | | |
| --- | --- | --- | --- | --- | --- | --- |
| Gene ID | Short discription | FPKM (6:00) | FPKM (7:00) | FPKM (10:00) | FPKM (14:00) | FPKM (18:00) |
| DN35955_c0_g1 | 60S ribosomal protein L10 | 262.5 | 220.3 | 187.6 | 259.9 | 319.7 |
| DN43515_c0_g3 | 60S ribosomal protein L12 | 80.2 | 69.5 | 58.6 | 77.7 | 90.1 |
| DN39009_c0_g1 | 60S ribosomal protein L13-2 | 307.0 | 242.9 | 183.3 | 231.9 | 317.3 |
| DN34366_c0_g3 | 60S ribosomal protein L14-2 | 140.8 | 125.7 | 113.3 | 128.0 | 151.8 |
| DN29521_c0_g1 | 50S ribosomal protein L15 | 40.2 | 42.1 | 34.5 | 46.1 | 53.5 |
| DN36809_c0_g1 | 60S ribosomal protein L18a | 122.4 | 106.6 | 102.1 | 116.6 | 150.4 |
| DN41431_c5_g1 | 60S ribosomal protein L18a-1 | 97.3 | 93.7 | 78.6 | 96.4 | 134.0 |
| DN46312_c1_g2 | 60S ribosomal protein L21-2 | 190.7 | 148.6 | 149.2 | 204.0 | 224.2 |
| DN43020_c5_g1 | 50S ribosomal protein L22 | 6.5 | 5.3 | 4.5 | 8.1 | 11.7 |
| DN42277_c0_g1 | 50S ribosomal protein L23 | 62.3 | 59.6 | 54.1 | 64.4 | 81.4 |
| DN46330_c2_g2 | 60S ribosomal protein L23A | 254.6 | 234.8 | 213.6 | 233.2 | 283.8 |
| DN37442_c2_g1 | 60S ribosomal protein L23A | 347.5 | 358.4 | 329.4 | 384.5 | 437.7 |
| DN45214_c0_g1 | 60S ribosomal protein L23 | 218.7 | 188.5 | 175.5 | 218.9 | 266.2 |
| DN29099_c0_g1 | 50S ribosomal protein L25 | 7.2 | 6.0 | 4.6 | 8.8 | 12.7 |
| DN42608_c0_g1 | 60S ribosomal protein L26-1 | 325.1 | 281.7 | 275.8 | 314.9 | 340.9 |
| DN36758_c0_g1 | 50S ribosomal protein L27 | 40.3 | 37.1 | 30.9 | 38.4 | 46.9 |
| DN30233_c0_g1 | 60S ribosomal protein L28-2 | 245.6 | 225.9 | 190.5 | 211.6 | 261.2 |
| DN30684_c0_g1 | 50S ribosomal protein L3 | 27.4 | 27.1 | 24.9 | 34.9 | 46.7 |
| DN35254_c0_g1 | 50S ribosomal protein L3-2 | 16.5 | 14.7 | 10.9 | 17.4 | 21.6 |
| DN31480_c0_g2 | 60S ribosomal protein L30 | 213.2 | 174.7 | 132.2 | 192.2 | 265.8 |
| DN41142_c0_g3 | 60S ribosomal protein L37 | 45.0 | 37.6 | 33.7 | 41.6 | 49.3 |
| DN34131_c3_g1 | 60S ribosomal protein L3 | 339.6 | 322.3 | 275.6 | 321.0 | 383.6 |
| DN30358_c1_g1 | 50S ribosomal protein L4 | 19.4 | 16.7 | 15.9 | 18.3 | 23.4 |
| DN32090_c0_g1 | 60S ribosomal protein L44 | 735.4 | 685.0 | 550.3 | 610.1 | 763.3 |
| DN30485_c0_g2 | 60S ribosomal protein L4-1 | 146.6 | 116.7 | 110.7 | 123.8 | 142.2 |
| DN33228_c1_g4 | 60S ribosomal protein L4-1 | 151.8 | 145.9 | 125.6 | 149.5 | 176.3 |
| DN31421_c0_g1 | 50S ribosomal protein L6 | 105.3 | 97.3 | 91.5 | 107.3 | 126.8 |
| DN42511_c0_g2 | 50S ribosomal protein L12 | 8.3 | 8.1 | 7.4 | 8.4 | 11.3 |
| DN41849_c0_g2 | 60S ribosomal protein L9 | 504.9 | 449.1 | 389.3 | 463.5 | 517.1 |
| DN36710_c0_g1 | 60S ribosomal protein L13 | 396.4 | 345.8 | 259.4 | 278.7 | 354.9 |
| DN34147_c0_g2 | 60S ribosomal protein L15 | 456.7 | 419.5 | 323.4 | 353.5 | 414.8 |
| DN44850_c0_g2 | 60S ribosomal protein L18-2 | 242.7 | 200.2 | 166.8 | 190.4 | 218.1 |
| DN47819_c3_g1 | 60S ribosomal protein L19-2 | 304.9 | 287.7 | 242.8 | 261.8 | 312.1 |
| DN36498_c2_g2 | 60S ribosomal protein L21-1 | 90.3 | 79.5 | 71.6 | 72.9 | 84.8 |
| DN38836_c4_g1 | 60S ribosomal protein L32-1 | 101.5 | 95.9 | 76.5 | 80.0 | 90.9 |
| DN35211_c1_g1 | 50S ribosomal protein L36 | 43.7 | 38.3 | 35.0 | 35.3 | 41.2 |
| DN36075_c0_g1 | 60S ribosomal protein L36-2 | 1046.3 | 910.3 | 652.4 | 715.0 | 923.6 |
| DN39959_c0_g2 | 60S ribosomal protein L8 | 290.9 | 260.2 | 218.8 | 241.3 | 277.6 |
| DN28760_c1_g1 | 30S ribosomal protein S21 | 20.8 | 21.5 | 14.1 | 17.1 | 32.0 |
| DN44927_c1_g1 | 30S ribosomal protein S6 | 73.6 | 69.1 | 61.1 | 71.2 | 75.1 |
| DN40200_c0_g2 | 30S ribosomal protein S18 | 50.7 | 42.8 | 29.6 | 41.8 | 50.6 |
| DN28293_c0_g2 | 40S ribosomal protein S11-2 | 236.6 | 222.8 | 193.0 | 216.6 | 256.5 |
| DN28293_c0_g1 | 40S ribosomal protein S11-3 | 193.7 | 183.1 | 165.1 | 193.6 | 208.1 |
| DN41516_c2_g1 | 40S ribosomal protein S12 | 347.2 | 326.0 | 269.3 | 316.4 | 374.9 |
| DN34496_c0_g2 | 40S ribosomal protein S14 | 21.0 | 17.2 | 15.2 | 19.7 | 21.9 |
| DN34989_c2_g2 | 30S ribosomal protein S15 | 10.4 | 11.7 | 8.9 | 13.2 | 13.6 |
| DN30918_c0_g1 | 40S ribosomal protein S15a-1 | 583.6 | 503.0 | 385.6 | 482.6 | 556.3 |
| DN41937_c1_g1 | 40S ribosomal protein S15 | 72.2 | 67.3 | 62.2 | 75.3 | 85.3 |
| DN37133_c0_g1 | 30S ribosomal protein S17 | 111.6 | 89.4 | 61.3 | 98.3 | 114.4 |
| DN34771_c1_g1 | 40S ribosomal protein S23 | 44.3 | 44.8 | 39.5 | 42.9 | 51.3 |
| DN31885_c0_g1 | 40S ribosomal protein S26-1 | 92.7 | 84.0 | 72.7 | 84.4 | 96.8 |
| DN39619_c1_g1 | 40S ribosomal protein S29 | 244.7 | 206.0 | 187.8 | 224.7 | 260.2 |
| DN34516_c0_g1 | 40S ribosomal protein S2-4 | 89.6 | 82.6 | 70.4 | 96.3 | 104.9 |
| DN34516_c0_g2 | 40S ribosomal protein S2-3 | 50.2 | 47.1 | 40.3 | 55.6 | 62.5 |
| DN35468_c1_g2 | 40S ribosomal protein S3a | 53.5 | 49.2 | 47.5 | 52.7 | 60.7 |
| DN39246_c1_g1 | 40S ribosomal protein S4 | 199.7 | 175.6 | 152.6 | 197.8 | 236.1 |
| DN39246_c1_g2 | 40S ribosomal protein S4-3 | 184.1 | 160.2 | 143.8 | 181.2 | 217.8 |
| DN32814_c0_g2 | 30S ribosomal protein S6 | 9.4 | 9.1 | 7.9 | 10.2 | 14.2 |
| DN33076_c3_g3 | 40S ribosomal protein S6 | 286.4 | 251.6 | 228.1 | 257.5 | 292.0 |
| DN33076_c3_g1 | 40S ribosomal protein S6 | 38.7 | 32.5 | 27.4 | 35.8 | 42.8 |
| DN28825_c1_g4 | 40S ribosomal protein S7 | 122.2 | 96.9 | 83.7 | 94.2 | 116.9 |
| DN45149_c0_g2 | 40S ribosomal protein SA | 82.9 | 83.8 | 71.6 | 78.8 | 98.3 |
| DN33413_c1_g2 | 40S ribosomal protein S5 | 120.6 | 111.6 | 96.3 | 106.3 | 120.8 |
| DN33413_c1_g1 | 40S ribosomal protein S5 | 201.2 | 184.6 | 159.0 | 178.4 | 198.0 |
| DN38773_c0_g2 | 40S ribosomal protein S16 | 58.7 | 50.9 | 34.9 | 41.6 | 51.4 |
| DN33554_c2_g1 | 40S ribosomal protein S18 | 183.2 | 152.4 | 126.7 | 137.3 | 162.3 |
| DN45562_c0_g1 | 40S ribosomal protein S20-2 | 337.1 | 321.2 | 273.5 | 282.9 | 351.0 |
| DN33604_c0_g1 | 40S ribosomal protein S23 | 417.9 | 403.7 | 336.1 | 365.1 | 425.0 |
| DN43015_c0_g1 | 40S ribosomal protein S24-2 | 509.2 | 478.0 | 344.9 | 381.7 | 472.9 |
| DN37589_c1_g2 | 40S ribosomal protein S27-2 | 706.2 | 585.4 | 492.6 | 524.4 | 610.8 |
| DN35468_c1_g1 | 40S ribosomal protein S3a | 414.2 | 389.0 | 369.8 | 366.2 | 421.3 |
| DN43690_c0_g1 | 40S ribosomal protein S8 | 222.9 | 206.7 | 146.6 | 164.6 | 195.8 |
| DN32501_c0_g2 | Eukaryotic translation initiation factor 2 | 77.2 | 70.1 | 65.3 | 68.4 | 77.8 |
| DN45971_c1_g1 | Eukaryotic translation initiation factor 3 | 111.3 | 114.6 | 107.8 | 112.3 | 128.7 |
| DN28531_c1_g1 | Eukaryotic translation initiation factor 3 | 62.5 | 57.5 | 48.2 | 58.8 | 65.4 |
| DN32875_c0_g4 | Eukaryotic translation initiation factor 3 | 70.2 | 68.1 | 58.2 | 64.9 | 72.1 |

| Table S7 Transcription factors (corresponding to Supp. Fig. 5E) | | | | | | |
| --- | --- | --- | --- | --- | --- | --- |
| Gene ID | **Short Discription** | **FPKM**  **(6:00)** | **FPKM**  **(7:00)** | **FPKM**  **(10:00)** | **FPKM**  **(14:00)** | **FPKM**  **(18:00)** |
| DN46071_c1_g1 | Homeobox protein knotted-1-like 6 | 258.8 | 214.4 | 190.5 | 220.5 | 235.2 |
| DN35594_c0_g2 | Homeobox protein knotted-1-like 3 | 12.4 | 13.2 | 10.5 | 11.2 | 16.1 |
| DN45686_c2_g2 | Homeobox-leucine zipper protein HAT5 | 101.6 | 96.3 | 79.5 | 97.9 | 134.7 |
| DN31824_c1_g2 | Homeobox-leucine zipper protein HAT5 | 44.6 | 39.7 | 32.0 | 42.2 | 59.4 |
| DN42650_c0_g5 | Dof zinc finger protein DOF1.4 | 39.4 | 42.4 | 34.2 | 39.7 | 41.6 |
| DN30987_c0_g1 | Dof zinc finger protein DOF2.4 | 72.4 | 83.3 | 71.1 | 79.4 | 87.9 |
| DN35974_c0_g1 | Zinc finger CCCH domain-containing protein 32 | 10.3 | 10.6 | 8.8 | 9.5 | 12.3 |
| DN38066_c2_g1 | Ethylene-responsive transcription factor ERF061 | 176.8 | 166.4 | 119.4 | 164.0 | 162.7 |
| DN39785_c0_g1 | Ethylene-responsive transcription factor ERF010 | 22.3 | 18.1 | 10.3 | 17.3 | 20.2 |
| DN44210_c7_g1 | Ethylene-responsive transcription factor ERF034 | 14.3 | 12.9 | 7.2 | 10.0 | 14.0 |
| DN35982_c1_g3 | Ethylene-responsive transcription factor RAP2-3 | 11.2 | 9.2 | 6.1 | 8.7 | 11.7 |
| DN38680_c1_g1 | Ethylene-responsive transcription factor CRF4 | 33.7 | 33.2 | 25.9 | 32.8 | 49.1 |
| DN32395_c0_g1 | AP2-like ethylene-responsive transcription factor | 4.6 | 3.3 | 2.5 | 3.2 | 4.5 |
| DN43243_c1_g4 | Protein indeterminate-domain 1 | 24.1 | 26.4 | 18.9 | 24.6 | 42.7 |
| DN37741_c0_g4 | Protein indeterminate-domain 1 | 16.0 | 16.4 | 12.9 | 18.9 | 25.8 |
| DN37741_c0_g1 | Protein indeterminate-domain 2 | 10.0 | 7.7 | 6.6 | 11.2 | 19.6 |
| DN45611_c0_g2 | NAC domain-containing protein 83 | 11.2 | 10.6 | 6.0 | 8.4 | 14.0 |
| DN29518_c1_g1 | NAC domain-containing protein 83 | 182.5 | 157.3 | 119.2 | 150.1 | 177.0 |
| DN46615_c0_g1 | NAC domain-containing protein 100 | 86.3 | 68.2 | 45.7 | 84.2 | 104.5 |
| DN45611_c1_g1 | NAC transcription factor 25 | 116.2 | 91.3 | 82.1 | 101.7 | 119.9 |
| DN39939_c1_g2 | NAC domain-containing protein 17-like isoform X1 | 69.4 | 59.3 | 55.8 | 62.0 | 69.9 |
| DN47620_c1_g1 | NAC domain-containing protein 48 | 160.4 | 107.4 | 79.5 | 112.6 | 109.4 |
| DN39641_c0_g1 | NAC domain-containing protein 2 | 200.7 | 158.3 | 110.2 | 145.7 | 183.7 |
| DN44051_c0_g4 | WRKY transcription factor 21 | 16.4 | 14.8 | 10.9 | 14.9 | 19.4 |
| DN39876_c1_g1 | WRKY transcription factor 75 | 58.9 | 47.2 | 28.9 | 46.1 | 65.5 |
| DN35314_c0_g1 | WRKY transcription factor 11 | 94.8 | 82.1 | 69.7 | 107.7 | 132.8 |
| DN46299_c1_g1 | WRKY transcription factor 42 | 6.8 | 5.2 | 4.8 | 6.4 | 8.9 |
| DN36051_c0_g1 | GATA transcription factor 21 | 10.9 | 8.3 | 9.0 | 12.5 | 22.6 |
| DN37250_c0_g2 | GATA transcription factor 16 | 1.6 | 1.5 | 1.3 | 2.1 | 2.7 |
| DN46138_c0_g1 | GATA transcription factor 22 | 37.2 | 41.1 | 28.1 | 32.9 | 53.3 |
| DN45560_c0_g1 | GATA transcription factor 7 | 17.2 | 10.6 | 5.6 | 7.4 | 9.5 |
| DN41405_c1_g2 | Transcription factor TGA4 | 14.9 | 12.5 | 7.5 | 9.5 | 19.9 |
| DN41405_c1_g1 | Transcription factor TGA4 | 66.6 | 56.3 | 43.2 | 63.0 | 77.6 |
| DN40668_c1_g2 | Heat stress transcription factor A-1 | 61.1 | 56.2 | 48.3 | 60.8 | 81.9 |
| DN36255_c0_g1 | Heat stress transcription factor A-1b | 16.6 | 12.6 | 5.7 | 9.5 | 20.9 |
| DN31263_c0_g1 | Probable transcription factor PosF21 | 14.9 | 14.3 | 14.2 | 15.6 | 19.1 |
| DN46380_c1_g2 | Trihelix transcription factor GT-1 | 18.5 | 18.3 | 12.5 | 19.1 | 19.3 |
| DN38164_c0_g1 | MADS-box transcription factor 16 | 810.2 | 857.4 | 755.6 | 793.9 | 880.8 |
| DN36360_c0_g3 | B3 domain-containing transcription factor | 1.7 | 1.1 | 0.7 | 0.9 | 2.8 |

**Table S8 NGS reads for waterlily RNA**

| Group | Read.No. | Clean.No. | rRNA.Read.No. | Percent.rRNA |
| --- | --- | --- | --- | --- |
| T1-1 | 19,064,210 | 18,768,057 | 253,542 | 1.35% |
| T1-2 | 26,793,547 | 26,416,474 | 208,761 | 0.79% |
| T1-3 | 31,102,986 | 30,640,258 | 515,338 | 1.68% |
| T2-1 | 27,523,147 | 27,034,432 | 244,160 | 0.90% |
| T2-2 | 22,869,361 | 22,514,820 | 156,355 | 0.69% |
| T2-3 | 34,877,447 | 34,324,482 | 498,243 | 1.45% |
| T3-1 | 28,709,249 | 28,223,818 | 151,785 | 0.54% |
| T3-2 | 22,469,332 | 22,083,265 | 111,928 | 0.51% |
| T3-3 | 21,689,137 | 21,336,557 | 148,808 | 0.70% |
| T4-1 | 21,513,940 | 21,137,717 | 118,648 | 0.56% |
| T4-2 | 20,441,360 | 20,129,292 | 130,589 | 0.65% |
| T4-3 | 19,095,326 | 18,778,722 | 145,067 | 0.77% |
| T5-1 | 19,427,535 | 19,137,934 | 131,292 | 0.69% |
| T5-2 | 21,398,893 | 21,086,230 | 195,550 | 0.93% |
| T5-3 | 24,088,524 | 23,740,190 | 140,857 | 0.59% |

**Table S9 Statistical result of waterlily RNA transcriptome assembly**

| Item | Gene number | Transcripts |
| --- | --- | --- |
| Total No. | 139,082 | 401,484 |
| Contig N10 | 4,601 | 4,695 |
| Contig N20 | 3,478 | 3,691 |
| Contig N30 | 2,720 | 3,032 |
| Contig N40 | 2,116 | 2,507 |
| Contig N50 | 1,622 | 2,062 |
| Median contig length | 450 | 773 |
| Average contig | 884 | 1,227 |
| Total assembled bases | 122,958,014 | 492,651,973 |
